# Duloxetine Inhibits Effects of MDMA (“Ecstasy") *In Vitro* and in Humans in a Randomized Placebo-Controlled Laboratory Study

**DOI:** 10.1371/journal.pone.0036476

**Published:** 2012-05-04

**Authors:** Cédric M. Hysek, Linda D. Simmler, Valentina G. Nicola, Nerina Vischer, Massimiliano Donzelli, Stephan Krähenbühl, Eric Grouzmann, Jörg Huwyler, Marius C. Hoener, Matthias E. Liechti

**Affiliations:** 1 Psychopharmacology Research Group, Division of Clinical Pharmacology and Toxicology, Department of Biomedicine and Department of Internal Medicine, University Hospital and University of Basel, Basel, Switzerland; 2 Divisions of Clinical Pharmacology and Toxicology, University Hospital, Lausanne, Switzerland; 3 Department of Pharmaceutical Sciences, University of Basel, Basel, Switzerland; 4 Pharmaceuticals Division, Neuroscience Research, F. Hoffmann-La Roche Ltd., Basel, Switzerland; Federal University of Rio de Janeiro, Brazil

## Abstract

This study assessed the effects of the serotonin (5-HT) and norepinephrine (NE) transporter inhibitor duloxetine on the effects of 3,4–methylenedioxy­methamphetamine (MDMA, ecstasy) *in vitro* and in 16 healthy subjects. The clinical study used a double-blind, randomized, placebo-controlled, four-session, crossover design. *In vitro,* duloxetine blocked the release of both 5-HT and NE by MDMA or by its metabolite 3,4-methylenedioxyamphetamine from transmitter-loaded human cells expressing the 5-HT or NE transporter. In humans, duloxetine inhibited the effects of MDMA including elevations in circulating NE, increases in blood pressure and heart rate, and the subjective drug effects. Duloxetine inhibited the pharmacodynamic response to MDMA despite an increase in duloxetine-associated elevations in plasma MDMA levels. The findings confirm the important role of MDMA-induced 5-HT and NE release in the psychotropic effects of MDMA. Duloxetine may be useful in the treatment of psychostimulant dependence.

**Trial Registration:**

Clinicaltrials.gov NCT00990067

## Introduction

Amphetamine derivatives, including 3,4-methylenedioxymethamphetamine (MDMA, “ecstasy") bind to monoamine transporters and potently release serotonin (5-hydroxytryptamine [5-HT]), norepinephrine (NE), and dopamine (DA) through the 5-HT (SERT), NE (NET), and DA (DAT) transporters, respectively [1], [2], [3], [4]. The pharmacological effect of MDMA can be blocked by monoamine transporter inhibitors. *In vitro*, the MDMA-induced release of NE, DA, or 5-HT from rat brain synaptosomes preloaded with monoamines is competitively inhibited by the monoamine transporter inhibitor indatraline [5], [6]. In humans, SERT inhibition reduced the psychotropic response to MDMA [7], [8], [9]. NET inhibition also attenuated the acute effects of MDMA [10] and amphetamine [11] in humans. In contrast, clonidine, which inhibits the vesicular release of NE, did not inhibit the effects of MDMA in humans [12]. Thus, the available evidence indicates that the MDMA-induced transporter-mediated release of 5-HT and NE appears to be involved in aspects of the acute subjective and cardiovascular responses to psychostimulants [2], [7], [10], [11]. However, the response to MDMA in humans was only moderately affected when either the SERT or NET was pharmacologically blocked [7], [10]. Therefore, we evaluated the effects of dual SERT and NET inhibition with duloxetine on the pharmacokinetics (PK) and pharmacodynamics (PD) of MDMA in humans. Duloxetine was used because it is the most potent and selective dual SERT and NET inhibitor, although it also inhibits the DAT with 10- to 100-fold lower potency compared with the SERT and NET [13], [14]. MDMA is mainly metabolized to 3,4-dihydroxymethamphetamine (HHMA) by cytochrome P450 (CYP) 2D6-mediated *O*-demethylation, followed by catechol-*O*-methyltransferase-catalyzed methylation to 4-hydroxy-3-methoxymethamphetamine (HMMA) [15]. Because duloxetine inhibits CYP 2D6 [16], we expected an increase in plasma MDMA concentrations after duloxetine pretreatment. MDMA is also *N*-demethylated to the active metabolite 3,4-mehthylenedioxyamphetamine (MDA). Whether the effects of MDA on 5-HT and NE release are inhibited by transporter inhibitors is unknown. Additionally, the inhibition of MDMA’s effect on 5-HT and NE release by duloxetine has not been studied. Therefore, we also assessed the effects of duloxetine on 5-HT and NE release induced by MDMA or MDA *in vitro* using cells that express the respective human transporters. We also sought to link the *in vitro* and *in vivo* data to provide additional insights into the differential modulatory role of 5-HT and NE in the effects of MDMA in humans. Because the data on monoamine transporter affinity and inhibition have mostly been derived from studies that used rat transporters [17], we investigated the binding and inhibition characteristics of the human monoamine transporters for MDMA, MDA, and duloxetine and the transporter inhibitors used in previous clinical studies [7], [8], [9], [10] and *in vitro* studies [5], [6]. Finally, we used an *ex vivo* binding assay to assess whether plasma samples taken from the drug-treated participants in the clinical study exhibit SERT, NET, and DAT-binding properties *ex vivo*.

The overall hypothesis of the present study was that duloxetine would potently bind to SERT and NET and block the MDMA- and MDA-induced transporter-mediated release of 5-HT and NE *in vitro* and markedly reduce the acute effects of MDMA *in vivo* in humans.

## Methods

### Clinical Study

**Figure 1:**
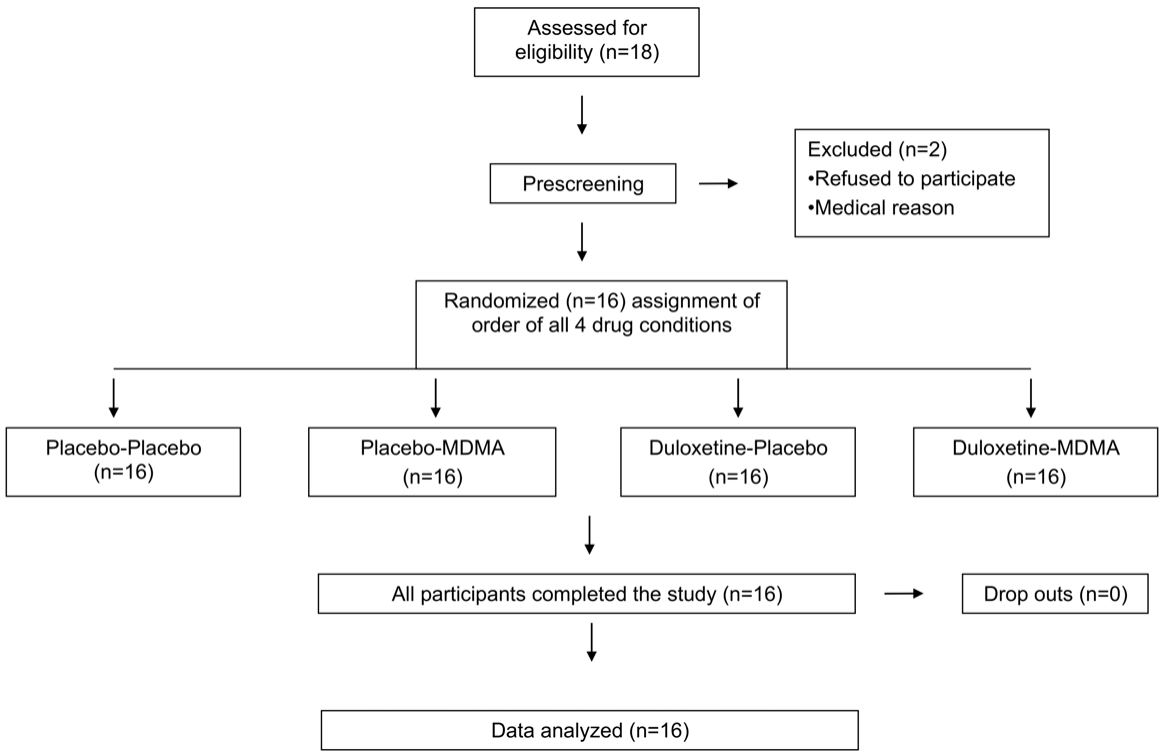
CONSORT flowchart.

The protocol for the clinical trial, the CONSORT checklist, and the CONSORT flowchart are available as supporting information; see Protocol S1, Checklist S1, and Figure 1. There were no changes to the protocol during the study.

### Ethics

The study was conducted in accordance with the Declaration of Helsinki and approved by the Ethics Committee of the Canton of Basel, Switzerland. All of the subjects provided written informed consent before participating in the study, and they were paid for their participation.

### Design

We used a double-blind, placebo-controlled, randomized, crossover design with four experiential conditions (placebo-placebo, duloxetine-placebo, placebo-MDMA, and duloxetine-MDMA) in a balanced order. The washout periods between the sessions were at least 10 days long.

### Participants

Sixteen healthy subjects (eight men, eight women) with a mean±SD age of 26.1±6.0 years participated in the study. The allocation to treatment order was performed by drawing from blocks of eight different balanced drug treatment sequences by a pharmacist not involved in the study. Each code was stored in a sealed envelope until the termination of the study. Data from all 16 subjects were available for the final analysis (Figure 1). The sample-size estimation showed that 13 subjects would be needed to detect a meaningful reduction of 20% of the MDMA drug effect by duloxetine with more than 80% power using a within-subjects study design. The exclusion criteria included the following; *(i)* age <18 or >45 years, (*ii*) pregnancy determined by a urine test before each session, *(iii)* body mass index <18.5 kg/m^2^ or >25 kg/m^2^, *(iv)* personal or family (first-degree relative) history of psychiatric disorder (determined by the structured clinical interview of Axis I and Axis II disorders according the *Diagnostic and Statistical Manual of Mental Disorders*, 4^th^ edition [18] supplemented by the SCL-90-R Symptom Checklist [19], [20] *(v)* regular use of medications, *(vi)* chronic or acute physical illness assessed by physical examination, electrocardiogram, standard hematological, and chemical blood analyses, *(vii)* smoking more than 10 cigarettes per day, *(viii)* a lifetime history of using illicit drugs more than five times with the exception of cannabis, *(ix)* illicit drug use within the last 2 months, and *(x)* illicit drug use during the study determined by urine tests conducted before the test sessions. None of the 16 subjects had used ecstasy previously. The subjects were asked to abstain from excessive alcohol consumption between the test sessions and limit their alcohol use to one glass on the day before the test session. All of the subjects were phenotyped for cytochrome P450 (CYP) 2D6 activity using dextromethorphan. Thirteen extensive, two intermediate, and one poor CYP 2D6 metabolizer were identified in the study. The female subjects were investigated during the follicular phase (day 2–14) of their menstrual cycle.

### Drugs

(± )MDMA hydrochloride (C_11_H_15_NO_2_, Lipomed, Arlesheim, Switzerland) was obtained from the Swiss Federal Office of Public Health and prepared as gelatin capsules (100 mg and 25 mg). Identical placebo (lactose) capsules were prepared. MDMA was administered in a single absolute dose of 125 mg that corresponded to an average dose of 1.87±0.36 mg/kg body weight. This dose of MDMA corresponds to a typical recreational dose of ecstasy, and comparable doses of MDMA have previously been used in controlled settings. Duloxetine (Cymbalta, Eli Lilly, Vernier, Switzerland) was prepared as 60 mg gelatine capsules, and identically looking placebo (lactose) capsules were similarly prepared. Duloxetine (120 mg) or placebo was administered twice 16 and 4 h before MDMA or placebo administration, respectively. The dose of the two administrations of duloxetine (120 mg/day on two separate days) was in the upper range of the chronic doses used clinically (60–120 mg/day). This dosing schedule was used to obtain high plasma concentrations of duloxetine similar to those reached with chronic administration of 60 mg/day. Drugs were administered without food.

### Assessments

#### Psychometric measures

The psychometric measures included Visual Analog Scales (VAS) [8], [10], the Adjective Mood Rating Scale (AMRS) [21], and 5-Dimensions of Altered States of Consciousness (5D-ASC) [22], [23]. The VASs included “any drug effect," “good drug effect," “bad drug effect," “drug liking," “drug high," “stimulated," “fear," “closeness to others," “talkative," and “open" [8], [10], [12], [24], [25]. The VASs were presented as 100 mm horizontal lines marked “not at all" on the left and “extremely" on the right. The VASs for “closeness to others," “open," and “talkative" were bidirectional (±50 mm). The VASs were administered 4 h before and 0, 0.33, 1, 1.5, 2, 2.5, 3, 3.5, 4, and 5 h after MDMA or placebo administration. The 60-item Likert-type scale of the short version of the AMRS [21] was administered 4 h before and 1.25, 2, and 5 h after MDMA or placebo administration. The AMRS contains subscales for activity, extroversion and introversion, well-being, emotional excitation, anxiety-depression, and dreaminess. The 5D-ASC rating scale measures alterations in mood, perception, experience of self in relation to the environment, and thought disorder. The 5D-ASC rating scale comprises five subscales or dimensions [22] and 11 lower-order scales [23]. The 5D-ASC dimension “oceanic boundlessness" (OB, 27 items) measures derealization and depersonalization associated with positive emotional states, ranging from heightened mood to euphoric exaltation. The corresponding lower-order scales include “experience of unity," “spiritual experience," “blissful state," and “insightfulness." The 5D-ASC dimension “anxious ego dissolution" (AED, 21 items) summarizes ego disintegration and loss of self-control phenomena, two phenomena associated with anxiety. The corresponding lower-order scales include “disembodiment," “impaired control of cognition," and “anxiety." The dimension “visionary restructuralization" (VR, 18 items) consists of the lower-order scales “complex imagery," “elementary imagery," “audiovisual synesthesia," and “changed meaning of percepts." Two other dimensions of the scale were not used in our study. The global ASC score was determined by adding the OB, AED, and VR scores. The 5D-ASC scale was administered 4 h after MDMA or placebo administration.

#### Physiologic measures

Physiologic measures were assessed repeatedly 4, 3, 2, and 1 h before and 0, 0.33, 0.66, 1, 1.5, 2, 2.5, 3, 4, 5, and 6 h after MDMA or placebo administration. Heart rate, systolic blood pressure, and diastolic blood pressure were measured using an OMRON M7 blood pressure monitor (OMRON Healthcare Europe, Hoofddorp, The Netherlands). Measures were taken twice per time point with an interval of 1 min, and the average was used for the analysis. Core (tympanic) temperature was assessed using a GENIUS 2 ear thermometer (Tyco Healthcare Group, Watertown, NY). The temperature of the room was maintained at 23.2±0.5°C. Adverse effects were assessed using the List of Complaints (LC) [26], which consists of 66 items that yield a total adverse effects score and reliably measure physical and general discomfort.

#### Plasma catecholamines and Pharmacokinetics (PK)

Blood samples to determine the concentrations of NE and epinephrine were collected 4 h before and 1 and 2 h after MDMA or placebo administration. The levels of free catecholamines (NE and epinephrine) were determined using high-performance liquid chromatography (HPLC) with an electrochemical detector as described previously [10]. Plasma concentrations of copeptin were also determined in this study as reported elsewhere [27]. Samples of whole blood for the determination of MDMA, MDA, HMMA, and duloxetine were collected into lithium heparin monovettes -4, 0, 0.33, 0.66, 1, 1.5, 2, 2.5, 3, 4, and 6 h after administration of MDMA or placebo. Plasma concentrations of MDMA, MDA, HMMA, and duloxetine were analyzed by HPLC coupled to a tandem mass spectrometer as described previously [12]. The assays were linear in the concentration ranges of 1–1000 ng/ml for MDMA and MDA, 1–500 ng/ml for HMMA, and 2.5–1000 ng/ml for duloxetine. The performance of the method was monitored using quality control (QC) samples at the lower limit of quantification (LLOQ) and at two or three QC concentrations. The interassay accuracy values for the QC samples ranged from 97.5% to 100% for MDMA, from 95.3% to 103% for MDA, from 91.1% to 106% for HMMA, and from 93.2% to 96.4% for duloxetine. The interassay precision values ranged from 2.8% to 8.0% for MDMA, from 3.8% to 10.5% for MDA, from 3.1% to 8.8% for HMMA, and from 4.7% to 9.3% for duloxetine. No hydrolysis was performed. Thus, the values for HMMA represent the drug concentrations of the non-conjugated metabolite. All blood samples were collected on ice and centrifuged within 10 min at 4°C. The plasma was then stored at –20°C until the analysis.

### In vitro Studies

#### Binding to monoamine transporters in vitro

Human embryonic kidney (HEK) 293 cells (Invitrogen, Zug, Switzerland) stably transfected with the human NET, SERT, or DAT as previously described [28] were cultured. The cells were collected and washed three times with phosphate-buffered saline (PBS). The pellets were frozen at –80°C. The pellets were then resuspended in 400 ml of 20 mM HEPES-NaOH, pH 7.4, that contained 10 mM EDTA at 4°C. After homogenization with a Polytron (Kinematica, Lucerne, Switzerland) at 10000 rotations per minute (rpm) for 15 s, the homogenates were centrifuged at 48000× g for 30 min at 4°C. Aliquots of the membrane stocks were frozen at –80°C. All assays were performed at least three times. The test compounds were diluted in 20 µl of binding buffer (252 mM NaCl, 5.4 mM KCl, 20 mM Na_2_HPO_4_, 3.52 mM KH_2_PO_4_, pH 7.4) and 10 point dilution curves were made and transferred to 96-well white polystyrene assay plates (Sigma-Aldrich, Buchs, Switzerland). *N*-methyl-^3^H-nisoxetine (∼87 C_i_/mmol, Perkin-Elmer) was the radioligand for the NET assay and had a dissociation constant (K_d_) of 9 nM. Fifty microliters of 12 nM [^3^H]-nisoxetine was added to each well of the assay plates, targeting a final [^3^H]-nisoxetine concentration of 3 nM. [^3^H]-citalopram (∼72 C_i_/mmol; Perkin-Elmer) was the radioligand for the SERT assay and had a K_d_ of 2.2 nM. Fifty microliters of 8 nM [^3^H]-citalopram was added to each well of the SERT assay plates, targeting a final [^3^H]-citalopram concentration of 2 nM. [^3^H]-WIN35,428 (∼86 C_i_/mmol; Perkin-Elmer) was the radioligand for the DAT assay and had a K_d_ of 12 nM. Fifty microliters of [^3^H]-WIN35,428 (∼40 nM concentration) was added to each well of the hDAT assay plates, targeting a final [^3^H]-WIN35428 concentration of 10 nM. Twenty microliters of binding buffer alone in the assay plate defined the total binding, whereas binding in the presence of 10 µM indatraline defined nonspecific binding. Frozen NET, SERT, or DAT membrane stocks were thawed and resuspended to a concentration of approximately 0.04 mg protein/ml binding buffer (1∶1 diluted in H_2_O) using a polytron tissue homogenizer. The membrane homogenates (40 µg/ml) were then lightly mixed for 5–30 min with polyvinyl toluene (PCT) wheat germ agglutinin-coated scintillation proximity assay (WGA-SPA; Amersham Biosciences) beads at 7.7 mg beads/ml homogenate. One hundred thirty microliters of the membrane/bead mixture were added to each well of the assay plate that contained radioligand and test compounds (final volume in each well, 200 µl) to start the assay, which was incubated for approximately 2 h at room temperature with agitation. The assay plates were then counted in the PVT SPA counting mode of a Packard Topcount. Fifty microliters of the [^3^H]-nisoxetine, [^3^H]-citalopram, or [^3^H]-WIN35428 stocks were counted in 5 ml of ReadySafe scintillation cocktail (Beckman Industries) on a Packard 1900CA liquid scintillation counter to determine the total counts added to the respective assays. Non-linear regression was used to fit the data to sigmoid curves and determine IC_50_ values for binding and uptake. K_i_ values for binding and uptake were calculated using the following Cheng-Prusoff equation: *K_i_ = IC_50/_(1+ [S]/K_m_).* [29].

#### Monoamine uptake in vitro

Two different methodological approaches were used to assess the effects of the drug on monoamine uptake. Method A used centrifugation through silicon oil, and method B used buffer to stop the reaction and wash the cells. *Method A:* The SERT, NET, and DAT functions were evaluated in human HEK 293 cells that stably expressed human SERT, NET, and DAT. The cells were grown in Dulbecco’s modified Eagle’s medium (Invitrogen, Zug, Switzerland) with 10% fetal bovine serum and 250 µg/ml geneticine. The cells (100 µl, 4×10^6^ cells/ml) were incubated for 10 min with 25 µl uptake buffer (9.99 mM L-glucose, 0.492 mM MgCl_2_, 4.56 mM KCl, 119.7 mM NaCl, 0.7 mM NaH_2_PO_4_, 1.295 mM NaH_2_PO_4_, 0.015 mM sodium bicarbonate, and 1 mg/ml ascorbic acid for [^3^H]-DA uptake) that contained various concentrations of inhibitor at 25°C. Fifty microliters of 5 nM (final concentration) [^3^H]-5-HT (80 C_i_/mmol; Anawa), [^3^H]-NE (14.8 C_i_/mmol; Perkin-Elmer), or [^3^H]-DA (13.8 C_i_/mmol; Perkin-Elmer) was added to start uptake. Uptake was stopped after 10 min, and radioactivity was measured as described below for 5-HT and NE release. Cell integrity after MDMA treatment was confirmed by the Toxilight toxicity assay (Lonza, Basel, Switzerland). The data were fit by non-linear regression, and K_m_, EC_50_, and E_max_ values were calculated using Prism (GraphPad, San Diego, CA). Preliminary experiments showed that the accumulation of 5-HT and NE by the cells was time-dependent and complete after 5 min for both 5-HT and NE, respectively. The 5-HT and NE transport velocity was concentration-dependent and could be described by Michaelis-Menten kinetics. The K_m_ values were 489±147 nM, 450±125 nM, and 1707±297 nM for 5-HT, NE, and DA, respectively. Nonspecific uptake was determined for each experiment in the presence of 10 µM fluoxetine for SERT cells, 10 µM nisoxetine for NET cells, and 10 µM mazindol for DAT cells and subtracted from the total counts to yield specific uptake. Nonspecific uptake was <10% of total uptake. *Method B:* Ligand potencies to inhibit [^3^H]-DA, [^3^H]-5-HT, and [^3^H]-NE uptake via the human DAT, SERT and NET recombinantly expressed in HEK 293 cells were determined. The cells were grown in Dulbecco’s modified Eagle’s medium (Invitrogen, Zug, Switzerland) with 10% fetal bovine serum and 250 µg/ml geneticine in cell culture flasks. One day before the experiment, the cells were seeded in a volume of 110 µl at a density of 0.3 million cells/ml in 96-well plates (Packard) and incubated at 37°C and 5% CO_2_ overnight. On the day of the uptake experiment, the 96-well plates that contained the cells were washed with Krebs Ringer bicarbonate buffer (Sigma-Aldrich, Buchs, Switzerland). Test compounds (100 µl, diluted in Krebs Ringer bicarbonate buffer) were added to the microtiter plates and incubated at 37°C for 30 min. Afterward, 50 µl [^3^H]-DA (35–54 C_i_/mmol; Perkin-Elmer; final concentration, 100 nM), [^3^H]-5-HT (28–100 C_i_/mmol; Perkin-Elmer; final concentration, 10 nM), or [^3^H]-NE (5.3–14 C_i_/mmol; Perkin-Elmer; final concentration, 100 nM) were added to DAT-, SERT-, and NET-containing cells, respectively, and incubated for 10 min at 37°C. Extracellular [^3^H]-DA, [^3^H]-5-HT, and [^3^H]-NE were removed, and the plates were washed twice with Krebs Ringer bicarbonate buffer. Nonspecific uptake was determined in the presence of 10 µM indatraline. Scintillant (Microscint 40, 250 µl) was dispensed to every well, and radioactivity was determined at least 1 h later on the Packard Topcount plate reader. The data were fit by non-linear regression, and the IC_50_ was calculated using Excel (Microsoft, Redmont, CA, USA). The compounds were tested at least three times. The K_m_ values were 1082 nM for [^3^H]-5-HT and >10000 nM for [^3^H]-DA and [^3^H]-NE.

#### 5-HT and NE release in vitro

Transporter-mediated MDMA- and MDA-induced 5-HT and NE release was evaluated using [^3^H]-5-HT- and [^3^H]-NE-preloaded HEK 293 cells that stably expressed human SERT and NET, respectively. The procedures were adapted from previous studies [2], [3]. SERT- or NET-expressing cells (100 µl, 4×10^6^ cells/ml) were incubated at 25°C for 10 min with 50 µl of 5 nM (final concentration) [^3^H]-5-HT or 10 nM [^3^H]-NE solutions, respectively. Steady-state load with radiolabeled substrate was reached within 5 min and remained stable for 60 min for both cell lines. Duloxetine or other transporter inhibitors (5 µl) were added after 10 min, and the release of [^3^H]-5-HT and [^3^H]-NE was then initiated after another 2 min by the addition of MDMA, MDA, or buffer (25 µl). The release reaction was stopped after 10 and 30 min for [^3^H]-5-HT and [^3^H]-NE, respectively. The release times were based on the evaluation of the release-over-time curves for MDMA and MDA. The release of [^3^H]-5-HT and [^3^H]-NE was complete within 5 and 25 min, respectively, when a new steady state was reached and maintained for 30 min. To stop the release reaction and wash the cells, 100 µl of the cell suspension was transferred to 0.5 ml microcentrifuge tubes that contained 50 µl of 3 M KOH and 200 µl silicon oil (1∶1 mixture of silicon oil types Ar20 and Ar200; Wacker Chemie, Munich, Germany) and centrifuged in a tabletop microfuge (Eppendorf, Basel, Switzerland) for 3 min at 13,200 rpm. This transports the cells through the silicon oil layer to the KOH layer, thereby separating the cells from the buffer, which remains on top of the silicon oil layer [30]. The centrifuge tubes were then transferred to liquid nitrogen. The amount of tracer that remained in the cells was quantified by cutting the frozen centrifuge tube above the KOH/oil interface and putting the tip of the tube with the cell pellet in a scintillation vial that contained 500 µl lysis buffer (0.05 M TRIS-HCl, 50 mM NaCl, 5 mM EDTA, and 1% Nonidet P-40 substitute in water). The samples were then shaken for 1 h on a rotary shaker, and 7 ml of scintillation fluid (Ultimagold, Perkin Elmer, Schwerzenbach, Switzerland) was added. Cell-associated radioactivity was then counted. The silicon oil assay allowed for the precise termination of the transport/release process and an effective cell wash. The experimental control condition (100% retained) was defined as the [^3^H]-5HT or [^3^H]-NE that remained in the cells when buffer and duloxetine were added without MDMA or MDA. A second control condition (100% release) was defined as the [^3^H]-5-HT or [^3^H]-NE released by 100 µM tyramine [6]. Data analysis using either of the two control conditions yielded similar results, and the data are presented as release expressed as the percentage of monoamine retained. Dose-response curves were generated using 9–11 concentrations of MDMA/MDA. Nonspecific binding/uptake was determined using preincubation with 10 µM fluoxetine for SERT cells and 10 µM nisoxetine for NET cells before incubation with radioligands and was <3% of total activity. All data points were derived from at least three independent experiments, each assayed in triplicate. The data were fit by non-linear regression, and EC_50_ and E_max_ values were calculated using Prism (GraphPad, San Diego, CA).

### Ex vivo Binding to Monoamine Transporters

Plasma samples for assessing *ex vivo* binding to monoamine transporters were collected 120 min after MDMA/placebo administration. We determined the potencies of the plasma to inhibit [^3^H]-nisoxetine, [^3^H]-citalopram, and [^3^H]-WIN35,428 binding to NET, SERT, and DAT, respectively, according to the method described previously [10]. IC_50_ values were calculated as a percentage of the plasma sample dilutions required to obtain 50% of the maximum effect. Indatraline (10 µM) in human plasma was used to achieve 100% inhibition. Undiluted plasma samples were set at 100%. Thus, an IC_50_ of 10% indicates that a 10-fold diluted plasma sample displaced 50% of the radioligand.

### Statistical Analyses

#### Pharmacodynamics

Clinical data values were transformed to differences from baseline. Peak effects (E_max_) were determined for repeated measures. E_max_ values were compared using General Linear Models repeated-measures analysis of variance, with drug as within-subject factor, using Statistica 6.0 software (StatSoft, Tulsa, OK). Tukey *post hoc* comparisons were performed based on significant main effects of treatment. Additional analyses of variance were performed, with period as factor to exclude period effects. Correlation analyses were performed using Pearson’s correlations. The criterion for significance was *p*<0.05. Mean arterial pressure (MAP) was calculated from diastolic blood pressure and systolic blood pressure using the following formula: *MAP = DBP+(SBP - DBP)/3*.

#### Pharmacokinetics

The plasma concentration data for MDMA, MDA, HMMA, and duloxetine were analyzed using non-compartmental methods. C_max_ and t_max_ were obtained directly from the observed concentration-time curves. The terminal elimination rate constant (λ_z_) was estimated by log-linear regression after semilogarithmic transformation of the data, using the last two to three data points of the terminal linear phase of the concentration-time curve of MDMA or duloxetine. Terminal elimination half-life (*t*_1/2_) was calculated using λ_z_ and the equation *t_1/2_ =  ln_2_/λ_z_*. The area under the plasma concentration-time curve up to 6 h (AUC_0-6h_) was calculated using the linear trapezoidal rule. The AUC_0–∞_ was determined by extrapolation of AUC_0–6h_ using λ_z_. The PK parameters were determined using the PK functions for Excel (Microsoft, Redmont, CA, USA). Plasma concentrations were only determined up to 6 h after MDMA administration because the aim of the study was to assess potential changes in MDMA plasma levels while relevant pharmacodynamic effects or MDMA were present. It was therefore not possible to determine t_1/2_ for HMMA and MDA because of their long t_1/2_, which would require sampling for an extended time.

*PK-PD modeling:* First, a soft-link PK-PD model was used to evaluate the *in vivo* relationship between the concentration of MDMA and subjective effect of the drug. The change in the VAS for any drug effect was used as the pharmacodynamic measure in each individual. Because we observed clockwise hysteresis in the effect-concentration relationship over time, we used PK-PD data pairs within the ascending part of the individual curves up to E_max_ or C_max_. Our estimate of E_max_, which should represent the maximal response portion of the dose-response curve, may already have been affected by tolerance. However, E_max_ values of 100% (scale maximum) or stable high values were reached by most subjects, indicating that tolerance was not an issue early in the effect-time curve. Based on the good brain penetration of MDMA and absence of a time lag, we assumed rapid equilibration between plasma and the central compartment (brain). A sigmoid E_max_ model was then fitted to the pooled data of all individuals: *E = E_max_ × C_p_^h/^(EC_50_^h^+C_p_^h^)*, in which *E* is the observed effect, *C_p_* indicates the MDMA plasma concentration, *EC_50_* indicates the plasma concentration at which 50% of the maximal effect is reached, *E_max_* is the maximal effect, and *h* is the Hill slope. The sigmoid E_max_ model provided a better fit than a simple E_max_ or linear model. Data pooling was used because only few data pairs were available per subject. Non-linear regression was used to obtain parameter estimates. Second, we also used a hard-link PK-PD model to predict *in vivo* PD effects based on the *in vitro* concentration-response data linked to the observed individual *in vivo* PK. The *in vitro* concentration-response relationship was described by a sigmoidal dose-response variable slope model fitted to the effects of MDMA on 5-HT or NE release using non-linear regression (Prism, GraphPad, San Diego, CA). The equation was the following: *E = E_max/_(1+10^(LogEC50-C)×h^)*, in which *C* denotes the concentration of MDMA in the assay, and *h* denotes the Hill slope. The *in vitro* effect-concentration relationship was determined for MDMA-induced 5-HT and NE release separately, and separate PD predictions were derived for each model. Similar to the soft-link PK-PD model, a single compartment PK model (plasma = brain concentration) was used, and only ascending PK or PD values were included. The *in vivo* data were linked to the PK of each individual, and a mean predicted effect-time curve was established.

## Results

### Pharmacodynamics (PD)

**Figure 2:**
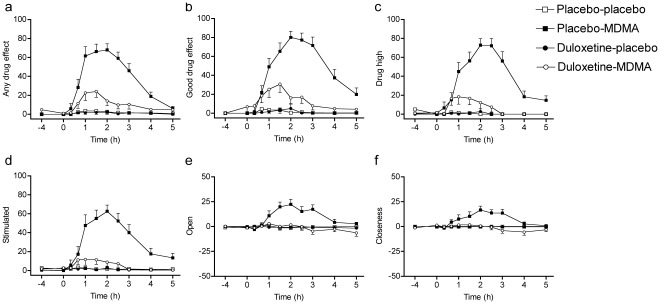
Duloxetine inhibited the psychotropic effects of MDMA. MDMA produced stimulant-like (**b–d**) and “entactogenic" (**e, f**) effects compared with placebo (*p*<0.001 for all scales). Duloxetine significantly inhibited MDMA-induced elevations in all of these subjective effects (**a–f**) (*p*<0.001 for all scales). Values are expressed as mean+SEM (*n*  = 16).

**Figure 3:**
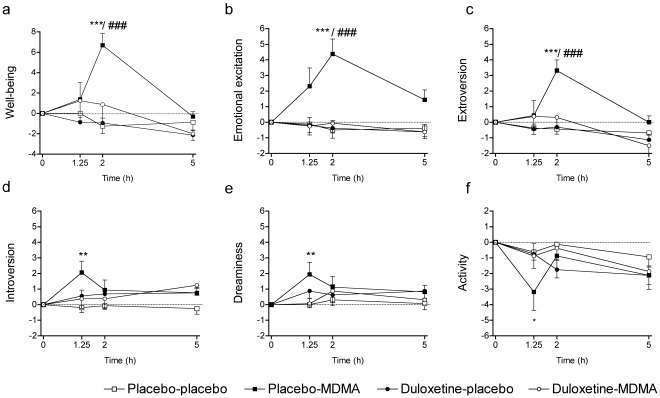
Duloxetine prevented the acute emotional effects of MDMA in the Adjective Mood Rating Scale. MDMA produced a state of well-being (a), emotional excitation (b), increased introversion at drug onset at 1.25 h (d), increased extroversion at 2 h (c), increased dreaminess (e), and decreased performance-oriented activity (f) (*p<0.05, **p<0.01, ***p<0.001, *vs*. placebo-placebo). Duloxetine prevented MDMA-induced elevations in well-being, emotional excitation, and extroversion (a-c) (^###^ *p*<0.001, placebo-MDMA *vs*. duloxetine-MDMA). Values are expressed as mean+SEM (*n*  = 16).

**Figure 4:**
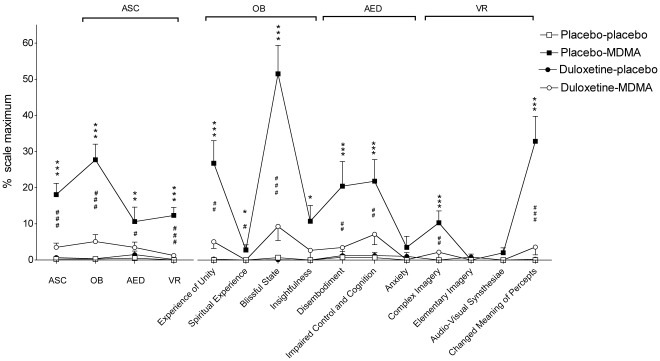
Duloxetine prevented the acute effects of MDMA in the Altered States of Consciousness (ASC) scale. MDMA significantly increased the ASC sum score, Oceanic Boundlessness (OB), Anxious Ego Dissolution (AED), and Visionary Restructuralization (VR) dimensions, and most of the subscales (*p<0.05, **p<0.01, ***p<0.001, placebo-placebo vs. placebo-MDMA). Duloxetine significantly reduced the effect of MDMA in all dimensions and subscales (^#^p<0.05, ^##^p<0.01, ^###^p<0.001, placebo-MDMA vs. duloxetine-MDMA). Values are expressed as mean+SEM (n  = 16).

**Figure 5:**
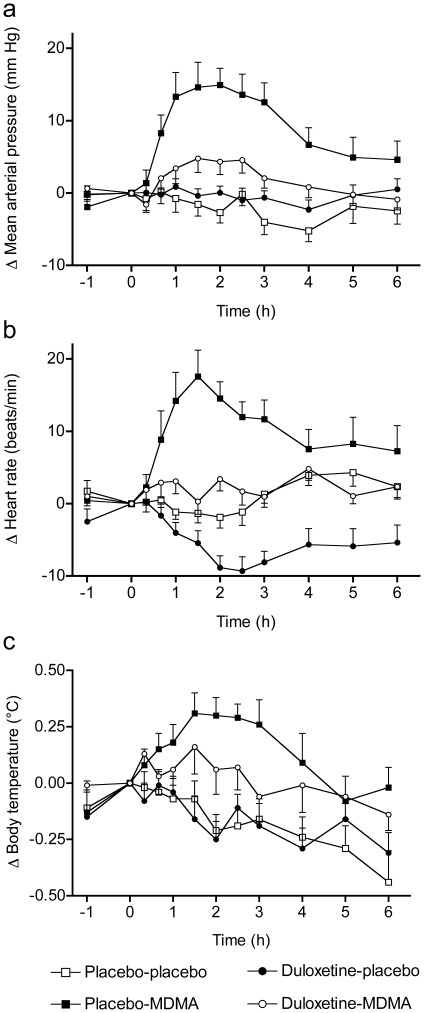
Duloxetine reduced the cardiostimulant response to MDMA. Duloxetine reduced the elevations in mean arterial blood pressure (**a**) and heart rate (**b**) in response to MDMA. Duloxetine also nonsignificantly lowered the MDMA-induced increase in body temperature (**c**). Values are expressed as mean+SEM of 16 subjects.

Duloxetine markedly reduced the psychotropic and cardiostimulant responses to MDMA in humans. Duloxetine decreased all aspects of MDMA’s subjective effects in the VASs [8], [10], including psychostimulant effects such as feelings of “good drug effects," “drug liking," “drug high," and “stimulation" (Table 1; Fig. 2b-d) but also so-called “entactogenic" or “empathogenic" MDMA-typical effects [31], [32] such as feelings of being “open," “closer to others," and more “talkative" (Table 1; Fig. 2e and f). In the AMRS [21], duloxetine prevented MDMA-induced increases in “well-being," “emotional excitation," and “extroversion" (Fig. 3). In the 5D-ASC [22], [23], duloxetine robustly reduced MDMA’s effects on the total ASC score (*p*<0.001) and in all three main dimensions of the scale (main effect of drug: *F*_3,45_ = 26.2, 32.6, 5.67, and 26.6 for ASC, OB, AED, and VR, respectively; all *p*<0.001; Fig. 4). Duloxetine prevented the MDMA-induced increase in circulating plasma NE levels, an endocrine marker for sympathetic system activation (Table 1), and reduced the blood pressure and heart rate response to MDMA (Table 1; Fig. 5). MDMA-induced increases in plasma NE at 60 min correlated with elevations in MAP (*r*  = 0.57, *p*<0.05) and increases in VAS scores for “good drug effects," “liking," “open" (r  = 0.65, 0.69, 0.77 and 0.63, respectively; all *p*<0.01), supporting the modulatory role of NE in these effects of MDMA. ANOVAs with period as factor showed no effect of treatment order, confirming the absence of period effects.

**Table 1:** Pharmacodynamic peak drug effects**.**

			Placebo-placebo	Duloxetine-placebo	Placebo-MDMA	Duloxetine-MDMA	*F*_3,45_ =	*p*<
Visual Analog Scales								
Any drug effect		E_max_	3.81±3.62	6.00±2.52^###^	86.69±3.57***	33.19±7.74*** ^###^	74.47	0.001
Good drug effect		E_max_	4.56±4.37	8.75±5.01^###^	89.38±4.67***	40.56±9.50*** ^###^	42.89	0.001
Drug liking		E_max_	4.13±4.06	7.56±4.43^###^	90.69±4.82***	38.38±8.91*** ^###^	52.60	0.001
Drug high		E_max_	1.94±1.94	4.81±2.93^###^	87.81±4.85***	28.94±9.35** ^###^	55.45	0.001
Stimulated		E_max_	4.13±1.94	5.13±2.45^###^	76.31±6.84***	22.25±7.65^###^	46.25	0.001
Open		E_max_	1.38±0.94	0.38±0.38^###^	32.16±4.29***	6.00±3.26^###^	36.88	0.001
Closeness		E_max_	0.00±0.00	0.00±0.00^###^	27.31±3.87***	4.63±2.49^###^	37.32	0.001
Talkative		E_max_	1.19±0.81	0.31±0.31^###^	28.81±5.12***	10.69±3.73^###^	21.13	0.001
Adjective Mood Rating Scale								
Well-being		E_max_	1.66±0.49	0.38±0.16^###^	7.06±1.01***	3.56±1.08^##^	18.0	0.001
Emotional excitation		E_max_	0.69±0.35	0.69±0.27^###^	4.94±0.97***	1.31±0.37^###^	14.7	0.001
Extroversion		E_max_	0.63±0.24	0.38±0.16^###^	3.50±0.61***	1.44±0.43^###^	17.5	0.001
Introversion		E_max_	0.38±1.56	1.13±0.30	2.62±0.65**	1.69±0.59	5.4	0.01
Dreaminess		E_max_	0.63±0.33	1.35±0.35	2.94±0.66**	1.81±0.48	4.1	0.05
Activity		E_min_	−1,88±0.50	−2.69±0.69	−4.69±1.04*	−2.81±0.78	2.6	0.06
Circulating catecholamines								
Epinephrine (nM)		E_max_	0.42±0.12	0.46±0.10	0.50±0.12	0.26±0.10		ns
Norepinephrine (nM)		E_max_	−0.22±0.13	−0.18±0.07^###^	0.44±0.12***	−0.19±0.10^###^	14.7	0.001
Physiologic effect								
SBP (mm Hg)		E_max_	8.56±1.75	6.19±1.42^###^	29.94±3.41***	10.94±1.58^###^	24.6	0.001
DPB (mm Hg)		E_max_	6.25±1.25	6.00±0.97^###^	22.13±2.08***	9.22±1.57^###^	23.3	0.001
MAP (mm Hg)		E_max_	5.80±1.27	5.11±1.01^###^	21.76±2.73***	8.54±1.46^###^	20.3	0.001
Heart rate (beats/min)		E_max_	9.19±1.29	5.06±1.27^###^	26.06±2.77***	11.09±1.55^###^	25.5	0.001
Body temperature (°C)		E_max_	0.23±0.04	0.19±0.04^###^	0.54±0.07**	0.39±0.08	7.3	0.001
List of Complaints (total score)								
Acute adverse effects		at 3 h	−0.06±0.52	−1.81±1.09^###^	5.56±1.72**	−1.25±1.49^##^	29.5	0.001
Sub-acute adverse effects		at 24 h	−1.00±0.58	−2.88±1.35^##^	3.88±1.09*	−0.38±1.32^#^	24.6	0.001
*Ex vivo* binding (IC_50%_)								
NET			>25	14.3±0.6*** ^##^	23.4±0.7	13.7±0.7*** ^###^	20.4	0.001
SERT			>25	1.5±0.2 *** ^###^	>25	1.4±0.2 *** ^###^	243.1	0.001
DAT			>25	>25	>25	>25		

Values are mean±SEM of changes from baseline of 16 subjects. *p<.05, **p<.01, and ***p<.001 vs. Placebo-placebo. ^#^p<.05, ^##^p<.01, ^###^p<.001 vs. Placebo-MDMA. SBP, systolic blood pressure; DBP, diastolic blood pressure; MAP, mean arterial pressure. IC50%, inhibition constant calculated as % of plasma sample dilution with undiluted plasma set as 100%; NET, norepinephrine transporter; SERT, SERT, serotonin transporter; DAT, dopamine transporter; ns, nonsignificant.

### Pharmacokinetics

**Figure 6:**
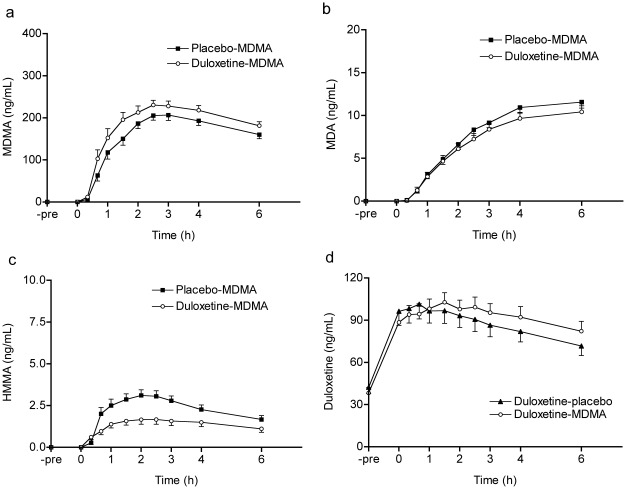
Duloxetine increased MDMA exposure. Pharmacokinetics of MDMA, MDA, HMMA, and duloxetine (**a–d**). Duloxetine was administered 16 h and 4 h before MDMA, which was administered at the 0 h time point. Duloxetine increased the C_max_ and AUC_0–6_ of MDMA (**a**), had no significant effect on MDA exposure (**b**), and decreased the C_max_ and AUC_0–6_ of HMMA (**c**). Plasma duloxetine concentrations were similar in the duloxetine-placebo and duloxetine-MDMA groups before MDMA administration (at –4 h and 0 h). Duloxetine concentrations increased 1 h after MDMA administration in the duloxetine-MDMA *vs*. duloxetine-placebo group (**d**). Values are expressed as mean±SEM of 16 subjects. MDMA, 3,4-methylenedioxymethamphetamine; MDA, 3,4-methylenedioxyamphetamine; HMMA, 4-hydroxy-3-methoxymethamphetamine.

The robust decrease in the PD response to MDMA after duloxetine was not the result of a pharmacokinetic interaction between duloxetine and MDMA because duloxetine increased exposure to MDMA. MDMA and duloxetine are both substrates and inhibitors of CYP 2D6 [16]. The moderate CYP 2D6 inhibitor duloxetine increased both the C_max_ and AUC_0-6h_ of the CYP 2D6 substrate MDMA by 16±4% (mean ± SEM; *F*_1,15_ = 12.64, *p*<0.01) and 18±5% (*F*_1,15_ = 8.95, *p*<0.01), respectively (Fig. 6 and Table 2). Duloxetine had no effect on exposure to MDA, the active metabolite of MDMA. Duloxetine decreased the C_max_ and AUC_0-6h_ of the inactive CYP 2D6-formed MDMA metabolite HMMA by 46±6% (*F*_1,15_ = 70.03, *p*<0.001) and 48±6% (*F*_1,15_ = 166.10, *p*<.001), respectively. Plasma duloxetine concentrations nonsignificantly increased beginning 1 h after MDMA administration (Fig. 5), consistent with the inhibitory effect of MDMA on duloxetine metabolism via CYP 2D6. Interindividual differences in CYP 2D6 activity also affected the PK of MDMA. Lower CYP 2D6 function (i.e., a lower dextromethorphan:dextrorphan urine concentration ratio) was associated with a longer t_1/2_ of MDMA (*r*  = 0.65, *p*<0.01).

**Table 2:** Pharmacokinetic parameters of MDMA, MDA, HMMA, and duloxetine.

		C_max_ (ng/ml)	T_max_ (h)	T_1/2_ (h)	AUC_0-6_ (ng/ml h)	AUC_(0-∞)_ (ng/ml h)
MDMA						
Placebo-MDMA		221.31±11.63	2.34±0.19	8.17±0.74	952.75±45.89	2908.55±275.64
Duloxetine-MDMA		253.63±13.60**	2.66±0.29	7.14±0.40	1106.87±57.22**	2915.28±154.27
MDA						
Placebo-MDMA		11.75±0.70	5.50±0.22	–	46.60±3.02	–
Duloxetine-MDMA		10.67±0.72	5.25±0.30	–	41.95±3.38	–
HMMA						
Placebo-MDMA		3.36±0.34	1.84±0.17	–	13.57±1.58	–
Duloxetine-MDMA		2.00±0.38***	1.89±0.25	–	8.14±1.45***	–
Duloxetine						
Duloxetine-placebo		106.77±10.25	5.14±0.29	10.97±1.04	799.88±74.40	1960.18±229.54
Duloxetine-MDMA		111.69±7.06	5.95±0.39	11.37±1.43	814.31±52.73	2189.45±297.99

C_max_, maximum plasma concentration; T_max_, time from drug administration to maximum plasma concentration; AUC_0-∞_, area under concentration-time curve extrapolated to infinity. HMMA, 4-hydroxy-3-methoxymethamphetamine; MDMA, 3,4-methylenedioxymethamphetamine; MDA, 3,4-methylenedioxyamphetamine. **p<.01, ***p<.001, vs. Placebo-MDMA. Values are mean±SEM (n  = 16).

### PK-PD Relationship

**Figure 7:**
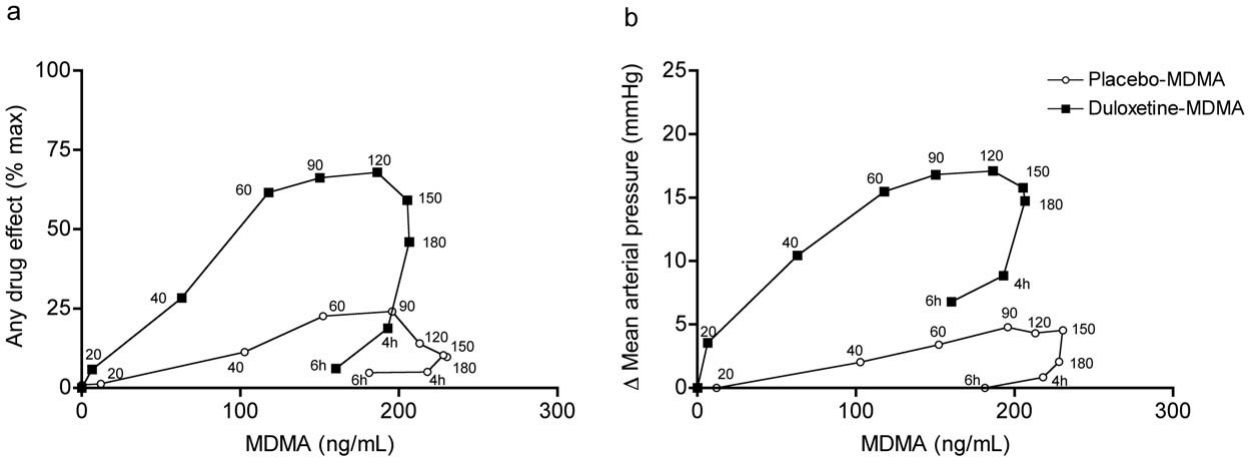
Pharmacokinetic-pharmacodynamic (PK-PD) relationship. MDMA effects are plotted against simultaneous MDMA plasma concentrations (**a, b**). The time of sampling is noted next to each point in minutes or hours after MDMA administration. The clockwise hysteresis indicates acute tolerance to the effects of MDMA. Duloxetine pretreatment markedly reduced physical and subjective responses to MDMA in the hysteresis loops (**a, b**).

Fig. 7 shows the mean PD effects of MDMA plotted against simultaneous plasma concentrations at the different time points (hysteresis loops). The increases in “any drug effect" (Fig. 7a) and MAP (Fig. 7b) returned to baseline within 6 h when MDMA concentrations were still high. This clockwise hysteresis indicates that a smaller MDMA effect was seen at a given plasma concentration later in time, indicating rapid acute pharmacodynamic tolerance, which was similarly described for cocaine [33]. Duloxetine robustly reduced the physical and subjective response to MDMA, but it increased exposure to MDMA, illustrated by the downward and rightward shift of the MDMA hysteresis loops (Fig. 7).

### Adverse Effects

MDMA produced adverse effects, such as sweating, difficulty concentrating, thirst, and lack of appetite, resulting in an increase in total LC scores at both 3 and 24 h after drug administration (Table 1). Duloxetine produced daytime somnolence and moderate insomnia. No severe adverse events were observed.

### In vitro Studies

**Figure 8:**
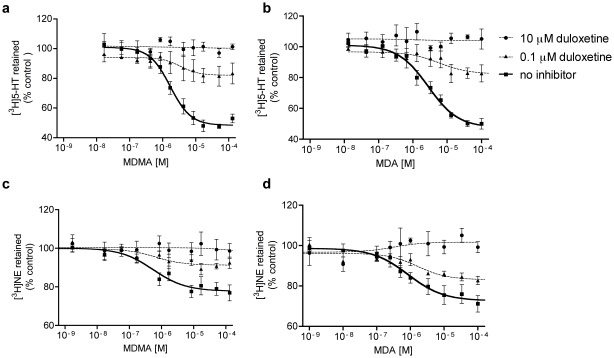
Duloxetine blocked MDMA- and MDA-induced 5-HT and NE efflux. Duloxetine inhibited SERT-mediated 5-HT release by MDMA (**a**) and MDA (**b**). Duloxetine also inhibited NET-mediated NE release by MDMA (**c**) and MDA (**d**). Values are expressed as mean ± SEM (*n*  = 3–6) of retained radiolabeled substrate following incubation with various concentrations of MDMA and MDA.

MDMA-induced 5-HT and NE release studies *in vitro*. MDMA was nonsignificantly more potent in releasing NE via NET than 5-HT via SERT (IC_50_ = 0.55 and 1.69 µM, respectively; Fig. 8; Table 3), consistent with earlier work that used human [3], [34] and rat [2] transporters. MDA similarly released monoamines with EC_50_ values of 0.85 and 2.77 µM for NE and 5-HT, respectively (Fig. 8; Table 3). Thus, both amphetamines were active transporter-mediated monoamine releasers and exhibited slightly higher potency at NET than SERT. Duloxetine potently inhibited the ability of MDMA and MDA to induce 5-HT release from SERT and NE release from NET cells (Fig. 8). Duloxetine (0.1 µM) decreased the E_max_ by approximately 50% and shifted the concentration-effect curves to the right, consistent with a mixed competitive and noncompetitive mode of inhibition. A high concentration of duloxetine (10 µM) completely blocked the effects of MDMA and MDA (Fig. 8). We then compared the inhibitory effect of duloxetine on MDMA-induced monoamine release to the inhibitory effects of the selective SERT inhibitor citalopram and selective NET inhibitor reboxetine, each of which have been shown to attenuate some of the effects of MDMA in humans [7], [10]. The potencies of duloxetine and citalopram to inhibit MDA- and MDMA-induced 5-HT release were similar (Fig. S1; Table 3). The potencies of duloxetine and reboxetine to block MDMA-induced NE release were also similar (Fig. S1; Table 3). These *in vitro* data indicate that duloxetine inhibited both SERT and NET similarly to citalopram and reboxetine, respectively.

**Table 3:** Inhibition of MDMA-induced 5-HT or NE release by different inhibitors.

	SERT		NET	
	EC_50_ (µM) (95% CI)	E_max_, % retained, (95% CI)	EC_50_ (µM) (95% CI)	E_max_, % retained, (95% CI)
MDMA alone	1.69 (1.07–2.66)	48 (42–55)	0.55 (0.17–1.81)	78 (73–82)
MDMA plus 0.1 µM duloxetine	3.51 (0.46–27)	82 (75–90)	0.59 (0.02–19)	90 (84–97)
MDMA plus 0.1 µM citalopram	3.17 (1.89–5.31)	72 (68–77)	na	na
MDMA plus 0.1 µM reboxetine	na	Na	3.35 (0.63–179)	78 (56–102)
MDA alone	2.77 (1.78–4.30)	48 (41–54)	0.85 (0.29–2.55)	73 (67–79)
MDA plus 0.1 µM duloxetine	6.86 (0.5–100)	83 (77–89)	2.06 (0.35–12.12)	80 (73–87)
MDA plus 0.1 µM citalopram	5.0 (1.28–19.6)	59 (44–75)	na	na

95% CI, 95% confidence interval; na, not assessed.

#### PK-PD and *in vitro-in vivo* relationship

**Figure 9:**
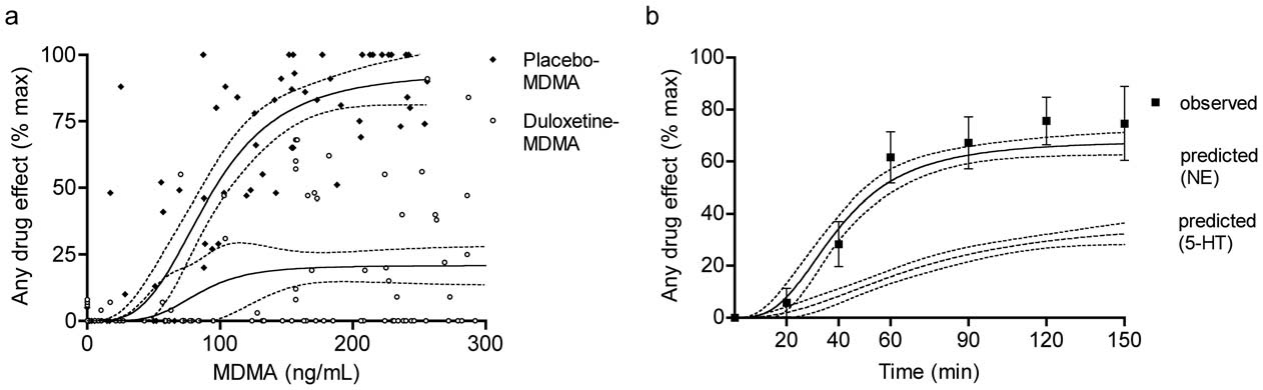
Pharmacokinetic-pharmacodynamic modeling. Duloxetine lowered E_max_ in the MDMA concentration-effect curve (**a**) with little effect on EC_50_, similar to the effect of MDMA on monoamine release *in vitro*. Diamonds and circles represent concentration-effect data pairs for ascending concentrations for placebo-MDMA and duloxetine-MDMA, respectively (**a**). The solid lines show the fit of a sigmoid E_max_ PD model to the observed PK data (**a**). Dashed lines indicate the 95% confidence interval (CI) of the estimation error (**a**). NE release predicted the observed subjective effect of MDMA *in vivo* (**b**). Predicted effects are shown as curves (mean ±95% CI) that represent the fit of the *in vitro* concentration-effect data to the 16 individual plasma concentration-time curves (**b**). Observed values are expressed as mean±SEM of 16 subjects (**b**). MDMA, 3,4-methylenedioxymethamphetamine; NE, norepinephrine; 5-HT, serotonin.

Duloxetine mainly affected the E_max_ of MDMA in the *in vivo* PK-PD relationship of MDMA (Fig. 9a) consistent with a primarily noncompetitive mode of inhibition and similar to the effect of duloxetine on monoamine release produced by MDMA *in vitro.* Duloxetine decreased the E_max_ from 93.8±7.3% to 20.8±4% for placebo-MDMA compared with duloxetine-MDMA, respectively. The EC_50_ values were 92.5±7.6 ng/mL (0.48 µM) and 83.8±25 ng/mL (0.43 µM) for placebo-MDMA and duloxetine-MDMA, respectively. The EC_50_ of the PK-PD curve of placebo-MDMA in humans was 74 ng/ml (0.38 µM), similar to the EC_50_ values of MDMA to release 5-HT and NE *in vitro*. The plasma concentrations of duloxetine (C_max_  = 112 ng/ml or 0.38 µM) were also in the range of the concentrations that reduced MDMA-induced 5-HT and NE release *in vitro*. To relate our *in vitro* data to the PD of MDMA in humans, we linked the concentration-effect relationship of the *in vitro* effect of MDMA on 5-HT and NE release to the individual concentration-time curves of our subjects (Fig. 9b). The observed effect-time curve for MDMA in humans was predicted well by the *in vitro* NE release model, assuming similar concentrations in plasma and brain and no time lag. The 5-HT release model fitted, but 2- to 10-fold higher MDMA concentrations in the brain than in plasma would be needed to obtain similar pharmacodynamic effects as NE. The higher potency of MDMA to release NE *vs*. 5-HT *in vitro* also predicted that NE release occurred at lower MDMA plasma and brain concentrations and therefore sooner after MDMA administration, playing a predominant role during the initial drug effect (i.e., rush, stimulant effect). 5-HT release becomes relatively more important later in time and predominantly mediates “entactogenic" effects, including feelings of being open and closer to others, that prevail later. The model predicted that the half-maximal effects would be reached at 40±2 min and 70±14 min for NE and 5-HT release, respectively (Fig. 9b). The observed half-maximal subjective drug effect of MDMA was reached 44±4 min after drug administration. At that time, the models predicted 4 (3–6)-fold higher NE release compared with 5-HT release, consistent with the view of a primary role for NE in the early effects of MDMA.

#### Monoamine transporter binding *in vitro*

The binding of MDMA and MDA to monoamine transporters was weak (Table 4) compared with the high potency of MDMA to release 5-HT and NE. The binding profile of MDMA was consistent with other binding studies that used human transporters [3] but different from studies that used rat transporters [17]. Duloxetine showed more than 100-fold higher affinity for both SERT and NET compared with the affinity of MDMA for these transporters in the same assay, supporting our approach of using duloxetine to prevent MDMA from interacting with SERT and NET (Table 4).

**Table 4:** Binding affinities to human monoamine transporters.

	SERT	NET	DAT
MDMA	13.3±0.47	22.4±14.6	6.52±2.24
MDA	18.7±2.76	17.8±4.06	26.4±4.24
Duloxetine	0.005±0.002	0.07±0.05	0.70±0.07
Reboxetine	0.24±0.02	0.015±0.01	16.2±4.91
Citalopram	0.005±0.001	5.06±3.00	21.4±10.5
Indatraline	0.02±0.008	0.03±0.02	0.01±0.01
Paroxetine	0.004±0.001	0.42±0.17	0.77±0.18

Values are mean±SD of K_i_ (µM) (n≥3). Radioligands were ^3^[H]citalopram, ^3^[H]nisoxetine, and ^3^[H]-WIN35,428 for SERT, NET, and DAT, respectively.

#### Monoamine uptake inhibition *in vitro*

MDMA inhibited NET three-fold more potently than SERT, consistent with previous studies that used human transporters [3], [35] but in contrast to data derived from mouse and rat transporters [17], [35], [36] (Table 5). MDA was equally potent to MDMA in inhibiting NET and SERT. Both MDMA and MDA showed low potency to inhibit DAT. Duloxetine was more potent in inhibiting SERT than NET (Table 5), which was expected [13]. Because the selective SERT inhibitor citalopram and selective NET inhibitor reboxetine have previously been shown to attenuate the psychological effects of MDMA [7], [10], we compared duloxetine with these inhibitors. Duloxetine exhibited similar potency as citalopram to inhibit SERT but 2- to 5-fold lower potency as reboxetine to inhibit NET (Table 5).

**Table 5:** Monoamine transport inhibition.

	SERT	NET	DAT
	K_i_ (µM) (95% CI)	K_i_ (µM) (95% CI)	K_i_ (µM) (95% CI)
MDMA*	1.40 (1.00–1.96)	0.470 (0.334–0.598)	16.7 (11.5–24)
MDA*	2.41 (1.49–3.92)	0.341 (0.253–0.461)	11 (7.5–17)
Duloxetine	0.050 (0.04–0.07)*	0.126 (0.099–0.161)*	2.26 (0.7–3.8)^#^
Reboxetine	2.07 (1.4–2.6)^#^	0.036 (0.030–0.044)*	16.4 (11.5–25.2)^#^
Citalopram*	0.045 (0.037–0.057)	>20	>20
Indatraline^#^	0.09 (0.06–0.12)	0.043 (0.03–0.06)	0.025 (0.01–0.04)
Paroxetine^#^	0.014 (0.01–0.02)	1.12 (0.03–1.7)	4.83 (2.4–7.3)

* method A; ^#^method B; 95% CI, 95% confidence interval; values are significantly different (p<0.05) if 95% CI do not operlap.

### Ex vivo Binding Studies

The ability of duloxetine to block monoamine transporters in our study was confirmed with an *ex vivo* assay, in which plasma from duloxetine-treated subjects inhibited *ex vivo* radioligand binding to SERT and NET but not DAT (Table 1). We also found a 10-fold higher affinity for SERT compared with NET, which was previously shown [13] and consistent with the *in vitro* profile of duloxetine. We calculated the duloxetine concentration in the plasma samples using the K_i_ values of duloxetine for SERT and NET binding (Table 2) and the IC_50_ values derived from the *ex vivo* binding in the duloxetine-placebo group (Table 1). The values (mean ± SE) obtained were 388±36 nM and 576±44 nM duloxetine using SERT and NET binding, respectively, which was well in agreement with the duloxetine plasma concentrations determined by LC-MS/MS (314±2.5 nM). Plasma from MDMA-treated subjects did not differ from placebo-treated subjects with regard to *ex vivo* radioligand binding to monoamine transporters (Table 1). This finding is consistent with the relatively low *in vitro* binding affinity of MDMA, which does not reflect the high pharmacological activity of the drug. Our assay assessed binding to the SERT and NET binding site for [^3^H]-citalopram and [^3^H]-nisoxetine, respectively. A possible explanation for the low affinity of MDMA in this assay could be a binding site for MDMA that is different from citalopram and nisoxetine at SERT and NET, respectively, consistent with the noncompetitive mode of inhibition of the MDMA-induced 5-HT and NE release by duloxetine.

## Discussion

The present study showed that the dual SERT and NET inhibitor duloxetine markedly decreased the psychotropic and cardiovascular responses to MDMA in human subjects, confirming and extending previous work with selective SERT [7], [8], [9] and NET [10] inhibitors. The inhibition of the effect of MDMA by duloxetine in humans was pronounced and primarily noncompetitive. *In vitro,* duloxetine similarly blocked the interactive effects of MDMA with SERT and NET to release 5-HT and NE. The present findings provide further support for a central role of SERT and NET as targets of MDMA with regard to its acute effects in humans. Previous clinical data indicated that 5-HT release primarily mediates the MDMA-typical “empathogenic" mood effects of MDMA [7], whereas NE release may be responsible for the stimulant and cardiovascular effects of the drug [10]. In the present study, dual inhibition of 5-HT and NE release robustly blocked both aspects of the MDMA effect, consistent with the role of both 5-HT and NE. The precise mode of interaction of amphetamine derivatives, including MDMA, with monoamine transporters remains to be elucidated and may involve the exchange of amphetamine with the transmitter, channel-like conformational changes of the transporter [37], or transporter internalization [38], [39], [40], MDMA is structurally similar to 5-HT, and a common binding site has been proposed in transmembrane domain 6 of SERT [41]. A distinct binding site was found for SERT inhibitors, including citalopram and fluoxetine, proximal to the 5-HT binding site [42]. Some SERT inhibitors may therefore allosterically inhibit the interaction between MDMA and SERT to release 5-HT. Consistent with these molecular data, our study showed that duloxetine inhibited MDMA-induced 5-HT release, NE release, and the response to MDMA in humans possibly according to a noncompetitive inhibition mode. Both our *in vitro* and *in vivo* findings may indicate acute allosteric inhibition of the effects of MDMA by duloxetine. Prior work with rat brain synaptosomes showed that indatraline competitively inhibited MDMA-induced 5-HT release [5]. However, later studies indicated that many SERT inhibitors also decreased the E_max_ for different monoamine releasers, suggesting unique transporter interactions for different inhibitor-releaser combinations [6]. This indicates that different SERT inhibitors may also more or less effectively reduce the effects of psychostimulants in humans. Nevertheless, several of the present findings indicate that the effect of duloxetine on the MDMA response was likely attributable to the dual inhibition of SERT and NET and not only the result of potent SERT inhibition alone. First, duloxetine blocked MDMA-induced NE release *in vitro* and MDMA-induced increases in plasma NE *in vivo*, similar to the selective NET inhibitor reboxetine [10]. Second, we documented, *ex vivo*, NET binding in plasma from duloxetine-treated subjects, and duloxetine has previously been shown to effectively inhibit NET in humans [13]. Third, potent and selective inhibition of SERT alone using citalopram in a single high dose [7], fluoxetine for 5 days [8], or paroxetine for 3 days [9] failed to block the effects of MDMA in humans to the extent seen here with dual SERT and NET inhibition. Conversely, selectively blocking NET alone also did not as effectively reduce the effects of MDMA in humans [10] as blocking both SERT and NET. The importance of NE as a modulator of the acute effects of MDMA is also supported by the fact that NE plasma levels after MDMA treatment in the present study correlated with the subjective effects and increases in blood pressure. Furthermore, we compared our *in vitro* 5-HT and NE release data to clinical data in humans and showed that the NE release link model better predicted the ascending subjective effects of MDMA in humans than the 5-HT release link model. A full assessment of the relative efficacy of SERT and NET inhibitors to prevent the effects of MDMA would require administration of SERT and NET inhibitors alone and in combination and dose-response studies. However, such studies were not ethically feasible because we did not want to expose our MDMA-naive subjects to more than two doses of MDMA in a crossover design.

The role of DA in the reinforcing effects of psychostimulants is well established, but unknown is whether DA is critical for the acute effects of MDMA. We found that MDMA exhibited higher affinity for DAT than NET or SERT *in vitro*. However, MDMA functionally exhibited significantly higher inhibition potency of the SERT and NET compared with DAT, respectively. MDMA is also more potent in releasing 5-HT and NE compared with DA *in vitro* [3], and the magnitude of 5-HT release exceeded DA release in the nucleus accumbens, striatum, and prefrontal cortex, assessed with *in vivo* microdialysis in rats [43]. DAT inhibition did not affect the acute response to MDMA in rhesus monkeys [44]. Additionally, the D_2_ dopamine receptor antagonist haloperidol only weakly attenuated MDMA-induced euphoria in humans and only at doses that produced significant dysphoria [45]. Whether DAT (NET) inhibitors, such as bupropion or methylphenidate, inhibit the effects of MDMA in humans remains to be tested. Duloxetine is a potent SERT and NET inhibitor but also weak DAT inhibitor [13], [46], which was confirmed in the present *in vitro* study. We cannot exclude the possibility that the relatively high dose of duloxetine used in the present study also inhibited MDMA-induced DA release. Notably, the present *ex vivo* binding studies further showed that the plasma from the subjects treated with duloxetine exhibited binding to SERT and NET but not DAT.

The transporter-independent vesicular release of monoamines could theoretically contribute to the mechanism of action of MDMA. We recently showed that this is not the case for NE because clonidine, which blocks transporter-independent vesicular NE release, did not alter the effects of MDMA in humans [12]. Additionally, MDMA did not directly stimulate the Ca^2+^-dependent vesicular release of DA [47]. Nevertheless, MDMA may indirectly stimulate the DA system and induce the vesicular release of DA by downstream 5-HT-DA or NE-DA system interactions. For example, 5-HT release by MDMA stimulates DA release via 5-HT_2_ receptor activation [48], and this indirect effect on the DA system is also prevented by SERT inhibition [49]. Thus, downstream DA system activation may be a contributing factor to MDMA-induced euphoria and the mechanism of action of psychostimulants in general, even when SERT and NET may be considered the primary pharmacological targets.

Finally, it is also possible that duloxetine induced adaptive effects on monoamine systems that reduced the response to MDMA in vivo. For example, decreases in SERT but not in NET binding sites were documented following chronic administration of duloxetine in rats [50].

In conclusion, the present study adds to a better understanding of the mechanism of action of MDMA in humans. The data support the roles of both NE and 5-HT in the acute effects of MDMA. The robust and almost complete prevention of the effects of MDMA by duloxetine suggests that dual transporter inhibitors may be useful in the prevention of the acute and long-term consequences of MDMA and potentially other psychostimulants in addicted subjects.

## Supporting Information

Figure S1 **Potency and efficacy of MDMA- and MDA-induced 5-HT and NE release inhibition by duloxetine, citalopram, and reboxetine.** Both duloxetine and citalopram inhibited MDMA-induced (a, c) and MDA-induced (b, d) 5-HT release *in vitro* with approximately similar potency and efficacy. The potency of duloxetine to block MDMA-induced NE release was also similar to the selective NET inhibitor reboxetine (e, f). EC_50_ and E_max_ values are shown in Table 3. Data points represent mean ± SEM.
(TIF)

Protocol S1 **Trial Protocol.**
(DOC)

Checklist S1 **CONSORT Checklist.**
(DOC)
