# Supplementary figures and images for: Duloxetine Inhibits Effects of MDMA (“Ecstasy") In Vitro and in Humans in a Randomized Placebo-Controlled Laboratory Study
Source: PLoS One. 2012 May 4;7(5):e36476. doi: 10.1371/journal.pone.0036476 (PMC3344887; doi:10.1371/journal.pone.0036476)

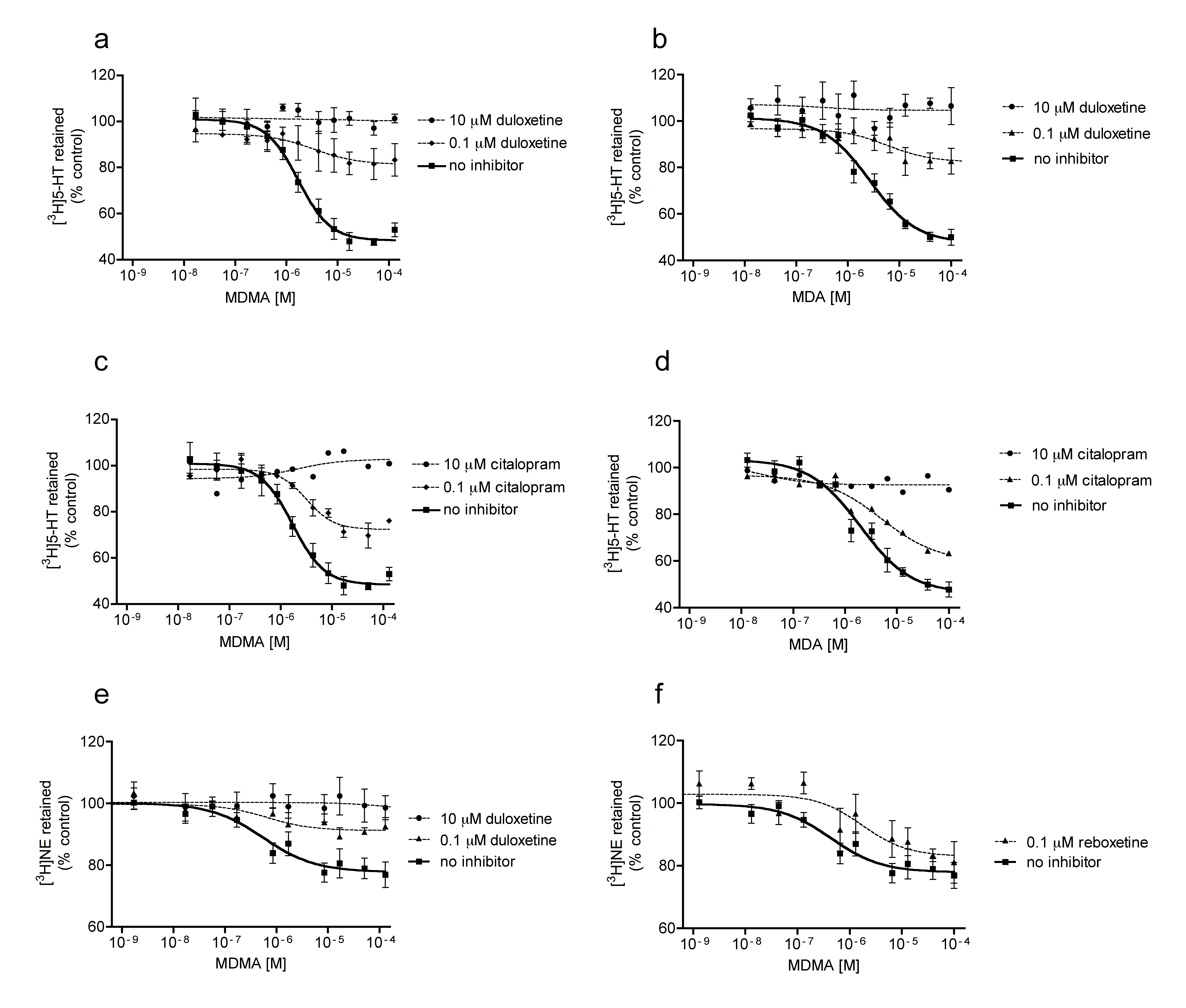

Supplement: Figure S1 — Potency and efficacy of MDMA- and MDA-induced 5-HT and NE release inhibition by duloxetine, citalopram, and reboxetine. Both duloxetine and citalopram inhibited MDMA-induced (a, c) and MDA-induced (b, d) 5-HT release in vitro with approximately similar potency and efficacy. The potency of duloxetine to block MDMA-induced NE release was also similar to the selective NET inhibitor reboxetine (e, f). EC50 and Emax values are shown in Table 3. Data points represent mean ± SEM. (TIF) [file pone.0036476.s001.tif]
