# Supplementary material for: Duloxetine Inhibits Effects of MDMA (“Ecstasy") In Vitro and in Humans in a Randomized Placebo-Controlled Laboratory Study
Source: PLoS One. 2012 May 4;7(5):e36476. doi: 10.1371/journal.pone.0036476 (PMC3344887; doi:10.1371/journal.pone.0036476)
Supplement: Protocol S1 — Trial Protocol. (DOC) [file pone.0036476.s002.doc]

**Pharmacological interaction between duloxetine and**

**3,4-Methylenedioxymethamphetamine (MDMA, Ecstasy)**

# SYNOPSIS

| Sponsor | Clinical Pharmacology & Toxicology, University Hospital Basel |
| --- | --- |
| Study title | Pharmacological interaction between duloxetine and 3,4-Methylenedioxy­­methamphetamine (MDMA, “Ecstasy”) |
|  |  |
| Study site | Divisions of Clinical Pharmacology & Toxicology, University Hospital Basel |
| Objectives | **Primary study endpoints**   1. Effect of duloxetine on the subjective response to MDMA   **Secondary study endpoints**   1. Effect of duloxetine on physiological responses to MDMA 2. Tolerability of MDMA and duloxetine 3. Effect of duloxetine on neuroendocrine effects of MDMA 4. Effects of duloxetine on pharmacokinetics of MDMA 5. Effect of MDMA on duloxetine pharmacokinetics |
| Design | Randomized, placebo-controlled, cross-over study |
| Analytics | Determination of plasma concentrations of MDMA, metabolites and duloxetine by GC-MS/LC-MS/MS |
| Subjects | 16 healthy volunteers (men or women), age: 19-45 years |
| Drugs | Duloxetine (Cymbalta®) and MDMA (3,4-methylene­­dioxy­­meth­­amphetamine) |
| Study dates | Start: 1.1.2010, End: 31.12.2010 |

# INDEX

[1 SYNOPSIS 0](#__RefHeading___Toc312139614)

[2 INDEX 1](#__RefHeading___Toc312139615)

[3 Abbreviations and Definitions 3](#__RefHeading___Toc312139616)

[4 SUMMARY 4](#__RefHeading___Toc312139617)

[5 INTRODUCTION 5](#__RefHeading___Toc312139618)

[6 SIGNIFICANCE 5](#__RefHeading___Toc312139619)

[7 BACKGROUND 5](#__RefHeading___Toc312139620)

[7.1 Pharmacology of MDMA 5](#__RefHeading___Toc312139621)

[7.2 Expected interactive effects of duloxetine and MDMA 6](#__RefHeading___Toc312139622)

[7.2.1 Pharmacodynamic interactions 6](#__RefHeading___Toc312139623)

[7.2.2 Pharmacokinetic interactions 7](#__RefHeading___Toc312139624)

[8 STUDY OBJECTIVES 7](#__RefHeading___Toc312139625)

[8.1 Primary study endpoints 7](#__RefHeading___Toc312139626)

[8.2 Secondary study endpoints 7](#__RefHeading___Toc312139627)

[9 HYPOTHESES 7](#__RefHeading___Toc312139628)

[9.1 Primary Hypotheses 7](#__RefHeading___Toc312139629)

[9.2 Secondary Hypotheses 7](#__RefHeading___Toc312139630)

[10 STUDY DESIGN AND METHODS 8](#__RefHeading___Toc312139631)

[10.1 Study design 8](#__RefHeading___Toc312139632)

[10.2 Study duration 8](#__RefHeading___Toc312139633)

[10.3 Study site 8](#__RefHeading___Toc312139634)

[10.4 Study population 8](#__RefHeading___Toc312139635)

[10.4.1 Recruitment 8](#__RefHeading___Toc312139636)

[10.4.2 Inclusion criteria 8](#__RefHeading___Toc312139637)

[10.4.3 Exclusion criteria 8](#__RefHeading___Toc312139638)

[11 STUDY PROCEDURES 9](#__RefHeading___Toc312139639)

[11.1 Schedule of Events 9](#__RefHeading___Toc312139640)

[11.2 Screening procedure 9](#__RefHeading___Toc312139641)

[11.2.1 Informed consent 9](#__RefHeading___Toc312139642)

[11.2.2 Physical health 9](#__RefHeading___Toc312139643)

[11.2.3 Mental health 9](#__RefHeading___Toc312139644)

[11.2.4 History of Drug use 9](#__RefHeading___Toc312139645)

[11.2.5 Screening laboratory tests 10](#__RefHeading___Toc312139646)

[11.2.6 CYP2D6 phenotyping 10](#__RefHeading___Toc312139647)

[11.2.7 Genotyping 10](#__RefHeading___Toc312139648)

[11.2.8 Personality 10](#__RefHeading___Toc312139649)

[11.2.9 Menstrual cycle phase in women 10](#__RefHeading___Toc312139650)

[11.3 Schedule of experimental session 10](#__RefHeading___Toc312139651)

[11.4 Assessments and Measures 11](#__RefHeading___Toc312139652)

[11.4.1 Psychometric assessments 11](#__RefHeading___Toc312139653)

[11.4.1.1 Visual Analogue Scales (VAS) 11](#__RefHeading___Toc312139654)

[11.4.1.2 Adjective mood rating scale (AMRS) 11](#__RefHeading___Toc312139655)

[11.4.1.3 State-trait anxiety inventory (STAI) 11](#__RefHeading___Toc312139656)

[11.4.1.4 Addiction Research Center Inventory (ARCI) 11](#__RefHeading___Toc312139657)

[11.4.1.5 Altered states of consciousness (5D-ASC) 12](#__RefHeading___Toc312139658)

[11.4.2 Physiological assessments 12](#__RefHeading___Toc312139659)

[11.4.2.1 Vital signs 12](#__RefHeading___Toc312139660)

[11.4.2.2 List of complaints (LC) and Adverse effects (AE) 12](#__RefHeading___Toc312139661)

[11.4.3 Cognitive and psychomotor function 12](#__RefHeading___Toc312139662)

[11.4.3.1 Reading the mind in the eyes test (RME) 12](#__RefHeading___Toc312139663)

[11.4.3.2 Choice reaction time test (CRTT) 12](#__RefHeading___Toc312139664)

[11.4.3.3 End of Session Questionnarie 13](#__RefHeading___Toc312139665)

[11.4.4 Neuroendocrine function 13](#__RefHeading___Toc312139666)

[11.4.5 Blood sample collection (pharmacokinetics) 13](#__RefHeading___Toc312139667)

[11.4.5.1 Amount of blood samples 13](#__RefHeading___Toc312139668)

[11.4.6 End of Study (EOS) visit 14](#__RefHeading___Toc312139669)

[11.4.6.1 EOS Examination 14](#__RefHeading___Toc312139670)

[11.4.6.2 EOS Questionnaire 14](#__RefHeading___Toc312139671)

[11.5 Study Drugs 14](#__RefHeading___Toc312139672)

[11.5.1 Duloxetine 14](#__RefHeading___Toc312139673)

[11.5.2 MDMA 15](#__RefHeading___Toc312139674)

[11.5.2.1 Chemistry, manufacturing, control 15](#__RefHeading___Toc312139675)

[11.5.2.2 Metabolism and Pharmacokinetics 15](#__RefHeading___Toc312139676)

[11.5.2.3 Dose selection 15](#__RefHeading___Toc312139677)

[11.5.3 Drug-Accountability, storage and return of study drugs 16](#__RefHeading___Toc312139678)

[11.5.4 Concomitant medications 16](#__RefHeading___Toc312139679)

[11.5.5 Randomization and Blinding 16](#__RefHeading___Toc312139680)

[11.5.6 Compliance 16](#__RefHeading___Toc312139681)

[12 Analytics 16](#__RefHeading___Toc312139682)

[12.1 MDMA and metabolites 16](#__RefHeading___Toc312139683)

[12.2 CYP2D6 Phenotype 16](#__RefHeading___Toc312139684)

[12.3 Cortisol and prolactin 16](#__RefHeading___Toc312139685)

[12.4 Catecholamines and metanephrines 16](#__RefHeading___Toc312139686)

[12.5 Copeptin 17](#__RefHeading___Toc312139687)

[12.6 Duloxetine 17](#__RefHeading___Toc312139688)

[13 Data analysis 17](#__RefHeading___Toc312139689)

[13.1 Sample size estimation 17](#__RefHeading___Toc312139690)

[13.2 Analysis of outcomes 17](#__RefHeading___Toc312139691)

[14 Protection of subjects 17](#__RefHeading___Toc312139692)

[14.1 Potential risks 17](#__RefHeading___Toc312139693)

[14.2 Monitoring of toxicity 18](#__RefHeading___Toc312139694)

[14.2.1 Safety definitions 18](#__RefHeading___Toc312139695)

[14.2.2 Documentation 19](#__RefHeading___Toc312139696)

[14.2.3 Adverse events (AE) documentation 19](#__RefHeading___Toc312139697)

[14.2.4 SAE and SUSAR reporting 20](#__RefHeading___Toc312139698)

[14.2.5 Medical follow-up of adverse events 20](#__RefHeading___Toc312139699)

[14.2.6 Monitoring for specific toxicity 20](#__RefHeading___Toc312139700)

[14.3 Risk-Benefit Assessment 21](#__RefHeading___Toc312139701)

[14.4 Ethical standards 21](#__RefHeading___Toc312139702)

[14.4.1 Institutional Review Board 21](#__RefHeading___Toc312139703)

[14.4.2 Protocol amendments 21](#__RefHeading___Toc312139704)

[14.4.3 Early study termination 22](#__RefHeading___Toc312139705)

[14.4.4 Insurance 22](#__RefHeading___Toc312139706)

[14.4.5 Compensation 22](#__RefHeading___Toc312139707)

[14.4.6 Premature withdrawal of subjects 22](#__RefHeading___Toc312139708)

[14.4.7 Replacement policy 22](#__RefHeading___Toc312139709)

[14.5 Legal authorizations 22](#__RefHeading___Toc312139710)

[14.6 Trial registration 22](#__RefHeading___Toc312139711)

[14.7 Study documentation and record keeping 22](#__RefHeading___Toc312139712)

[14.8 Quality control and quality assurance 23](#__RefHeading___Toc312139713)

[14.8.1 Training of personnel and SOPs 23](#__RefHeading___Toc312139714)

[14.8.2 Monitoring 23](#__RefHeading___Toc312139715)

[14.8.3 Direct access to source data 23](#__RefHeading___Toc312139716)

[14.8.4 Inspection 23](#__RefHeading___Toc312139717)

[14.9 Confidentiality of trial documents and subject records 23](#__RefHeading___Toc312139718)

[15 Time plan 23](#__RefHeading___Toc312139719)

[16 Publications 23](#__RefHeading___Toc312139720)

[17 Budget and Funding 24](#__RefHeading___Toc312139721)

[18 Certifications of principal investigator 24](#__RefHeading___Toc312139722)

[19 Conflict of interest 24](#__RefHeading___Toc312139723)

[20 Research Environment 24](#__RefHeading___Toc312139724)

[20.1 Study site 24](#__RefHeading___Toc312139725)

[21 References 24](#__RefHeading___Toc312139726)

# Abbreviations and Definitions

ASAT aspartate transferase

ALAT alanine aminotransferase

AE adverse event

ANOVA analysis of variance

AMRS adjective mood rating scale

ARCI addiction research center inventory

AUC area under the concentration time curve

BAG Bundesamt für Gesundheitswesen = Swiss Federal Office of Public Health

BP blood pressure

CHF Swiss francs

CRF case report form

CRTT choice reaction time test

CRC clinical research center

CRP C-reactive proteine

CYP cytochrome P450 enzyme

C_max_ maximum concentration

Cl chloride

Ca calcium

5D-ASC 5 dimensions of altered states of consciousness

EWL Eigenschaftswörterliste = AMRS

Ecstasy may contain MDMA or other substances at various amounts

EKBB Ethische Kommission beider Basel

ECG electrocardigram

EOS end of study

GCP good clinical practice

GC-MS gas chromatography-mass spectrometry

GMP good manufacturing practice

γ-GT gamma-glutamyl transferase

HPLC high-pressure liquid chromatography

5-HT serotonin

HR heart rate

HMA 4-hydroxy-3-methoxyamphetamine

HMMA 4-hydroxy-3-mehoxymethamphetamine

5-HIAA 5-hydroxy indole acetic acid

IRB institutional ethics board = EKBB

K potassium

LSD lysergic acid diethylamine

LC list of complaints

LDH laccate dehydrogenase

LC-MS liquid chromatography-mass spectroscopy

MDMA 3,4-Methylenedioxymethamphetamine

MDA 3,4-methylenedioxyamphetamine

MD medical doctor

NE norepinephrine = noradrenaline

Na sodium

P phosphorus

PK pharmacokinetic

PI principal investigator

P_sys_ systolic blood pressure

PhD Philosophical doctor (Dr. Phil. II)

THC tetrahydrocannabinol

SAE serious adverse event

SSRI selective serotonin uptake inhibitor

STAI state-trait anxiety inventory

SD standard deviation

T_1/2_ Plasma eliminiation half-life

T_max_ time to reach C_max_

VAS visual analogue scale

# SUMMARY

3,4-methylenedioxymethamphetamine (MDMA, “ecstasy”) is widely used by young people for its euphoric effects. MDMA releases serotonin (5-HT), norepinephrine (NE), and dopamine through an interaction with the corresponding presynaptic monoamine uptake transporter. 5-HT transport inhibitors block MDMA-induced 5-HT release in vitro or in animals and also attenuate the subjective and cardiovascular response to MDMA in humans. NE transport inhibitors similarly prevent the MDMA-induced release of NE in cell assays and attenuate behavioral effects of MDMA in animals. Effects of the NE transporter inhibitor reboxetine on the response to MDMA in humans are currently investigated. Here we suggest evaluating effects of pretreatment with the combined 5-HT and NE transport blocker duloxetine on the pharmacodynamics and pharmacokinetics of MDMA. The study will use a randomized double-blind cross-over design with four experimental sessions. Duloxetine (120 mg) or placebo will be administered 16 h and 4 h before the administration of MDMA (125 mg) or placebo to 16 healthy volunteers. Subjective and cardiovascular responses and plasma samples for pharmacokinetics will be repeatedly assessed throughout the experiments. We hypothesize that duloxetine will attenuate the subjective and cardiovascular response to MDMA.

**Key words:** MDMA, 3,4-Methylenedioxymethamphetamine, Ecstasy, norepinephrine, reboxetine, pharmacokinetics

# INTRODUCTION

3,4-methylenedioxymethamphetamine (MDMA, “ecstasy”) is widely used by young people for its euphoric effects. MDMA acts at the presynaptic monoamine transporter and releases serotonin (5-HT), dopamine (DA), and norepinephrine (NE) by reversing the transport of these monoamines. While the neurochemical effects of MDMA have been well studied in preclinical models, it is less clear how the neurochemistry translates into to the psychotropic and physiological effects in humans. Here, we suggest evaluating the functional role of 5-HT and NE in the pharmacology of MDMA in humans. We plan to test the effects of pretreatment with the selective and potent dual NE and 5-HT transport inhibitor duloxetine on the pharmacodynamics and pharmacokinetics of MDMA in healthy human volunteers. We hypothesize that duloxetine will attenuate subjective and cardiovascular responses to MDMA. Such a result would indicate that 5-HT and NE are critically involved in the pharmacology of MDMA and may provide helpful in the use and development of treatments for Ecstasy intoxications. The study shall take place in the Clinical Research Centre (CRC) of the Clinical Pharmacology Unit of the University Hospital of Basel.

# SIGNIFICANCE

Use of stimulant drugs including Ecstasy is highly prevalent in our society. In Switzerland, 2.2% of 15-39 year olds reported having used Ecstasy at least once [1]. Besides from being abused as a recreational drug, MDMA, the active substance usually found in Ecstasy pills, is being evaluated as a treatment for post-traumatic stress disorder in several countries including Switzerland. MDMA has also been suggested to be useful to treat and study mood disorders due to its potential to increase empathy and rapidly elevate mood [2, 3]. Ecstasy use can result in adverse and potentially fatal medical complications [4, 5]. Serious toxicity due to Ecstasy use is relatively rare but may be seen in up to ten percent of patients presenting to Emergency Departments with medical problems due to Ecstasy use [4]. Extensive Ecstasy use has also been associated with neurotoxic effects to serotonergic brain neurons [6]. Because MDMA is widely used, a better understanding of its pharmacology and toxicology is warranted.

# BACKGROUND

## Pharmacology of MDMA

In animals, MDMA releases presynaptic 5-HT, NE and to a lesser extent DA [7-9]. The MDMA-induced release of these monoamines is thought to be due to reverse-transport of monoamines through the corresponding uptake transporter. By blocking the interaction of MDMA with the 5-HT uptake site, 5-HT uptake inhibitors (SSRIs) decrease 5-HT release [9, 10] and the behavioral response to MDMA in animals [11, 12]. In humans, SSRIs including citalopram [13-15], fluoxetine [16], and paroxetine [17] similarly attenuated the subjective effects of MDMA. These findings confirm that the interaction of MDMA with the 5-HT uptake site is an important mode of action of this drug of abuse. However, blockade of MDMA-induced 5-HT release or postsynaptic antagonism of serotonin or dopamine receptors only partly blocked MDMA’s effects and other neurotransmitter systems are likely to contribute to the pharmacological response to MDMA [18]. In particular, recent evidence from in vitro and animal studies indicates an important role for NE in the pharmacology of MDMA. MDMA exhibits higher affinity for the NE transporter compared to the human 5-HT or DA uptake site [8, 19]. In addition, MDMA was more potent in releasing NE than 5-HT or DA from monoamine preloaded human embryonic kidney cells transfected with the corresponding human monoamine transporters [19]. The NE uptake transporter inhibitor desipramine reversed acute cognitive effects of MDMA in resus monkeys [20]. Further, doses of stimulants, which produce amphetamine-type subjective effects in humans, correlated with their potency to release NE and not DA release [8]. NE also plays an important role in mediating peripheral effects of MDMA. MDMA dramatically increases circulating plasma levels of NE [21]. The postsynaptic adrenergic α_1_ receptor antagonist prazosin reversed MDMA-associated locomotor stimulation in rats [22]. In addition, blockade of the effects of NE at postsynaptic α_1_ and β_1-3_ receptors reversed the hyperthermic response to MDMA in rats [21]. Further, blockade of the NE transporter abolished contraction of the rat aorta produced by 4-methylthioampethamine [23], a compound with a similar pharmacology to MDMA. Clinically, MDMA stimulates the sympathetic nervous system and increases heart rate, blood pressure and body temperature in controlled settings [15, 24]. Serious adverse effects of uncontrolled Ecstasy use also include hypertensive and hyperthermic reactions that are likely to be mediated by an activation of the adrenergic system by MDMA [5]. Together, the preclinical and clinical findings suggest that 5-HT and NE mediate most of the psychotropic and cardiovascular effects of MDMA in humans.

## Expected interactive effects of duloxetine and MDMA

### Pharmacodynamic interactions

Duloxetine (Cymbalta) is a potent inhibitor of the 5-HT and NE transporter [25-27]. In radioligand binding assays utilizing rat cortex synaptosomes, duloxetine possesses high affinity for both the NE and 5-HT transporter with K_i_ values of 2.1 and 0.53 nM, respectively [27]. Duloxetine is selective for the NE and 5-HT system and does not directly modulate the DA system. The affinity of duloxetine for the 5-HT transporter is similar to that of citalopram for this transporter (1.8 nM). We have previously shown that citalopram pretreatment significantly reduced the psychological and cardiovascular response to MDMA in healthy subjects [13, 15]. Because duloxetine is at least equally potent in blocking the 5-HT transporter compare to citalopram and also blocks the NE transporter with high potency we expect that duloxetine will be at least equally effective in decreasing the response to MDMA as citalopram. Actually, we hypothesize that duloxetine will be considerably more effective in reducing the cardiovascular effects of MDMA due to its additional binding to the NE transporter which is expected to prevent MDMA from interacting with the NE transporter to release NE [19]. The 5-HT and NE transport inhibition by duloxetine results in slight increases in brain 5-HT and NE [26]. In the present study, however, the ability of duloxetine to bind to the NE and 5-HT transporter is used to block the effects of MDMA which releases 5-HT and NE through an interaction with the NE and 5-HT transporter [8]. In preclinical studies, duloxetine prevented effects of different amphetamine derivatives with structural similarity to MDMA [26]. For example, duloxetine was shown to antagonize depletion of brain 5-HT by p-chloroamphetamine or of brain or heart NE by 6-HO-dopamine [26, 28]. No preclinical studies have studied the interaction of duloxetine and MDMA at the level of the NE or 5-HT transporter. Also no previous clinical study has addressed whether dual blockade of NE and 5-HT transport effectively blocks effects of stimulant drugs in humans. However, several compounds that block either 5-HT or NE transport have consistently been shown to block MDMA-induced release of NE or 5-HT in various assays [7, 8, 20, 29]. As noted above, 5-HT blockade reduced responses to MDMA in humans. Further, NE transport blockade with desipramine decreased acute cognitive effects of MDMA in resus monkeys [20]. In addition, recent clinical studies found that the NE transport blocker atomoxetine attenuated cocaine-induced systolic blood pressure increases [30] and cardiovascular and subjective responses to d-amphetamine [31]. These clinical data indicate that pretreatment with an NE transport antagonist decreases the cardiovascular stimulant effects of cocaine or d-amphetamine that have a very similar pharmacological action to MDMA. Together the data indicate that duloxetine will decrease the response to MDMA in healthy subjects.

### Pharmacokinetic interactions

Duloxetine is a substrate and inhibitor of CYP 2D6 [32] similar to MDMA [33]. Duloxetine may therefore increase MDMA plasma levels and vice versa. However, because MDMA itself inhibits CYP 2D6 (autoinhibition) the additive effect of duloxetine is expected to be small. Pretreatment with paroxetine which is considerably more potent in inhibiting CYP 2D6 than duloxetine increased MDMA plasma levels by only 30% [17]. Thus, we do not expect a relevant increase in MDMA C_max_ or AUC when duloxetine is administered before MDMA (<30% increase in C_max_ or AUC). On the other hand, MDMA may moderately increase C_max_ and AUC of duloxetine thereby enhancing the pharmacodynamic interaction. This potential effect is not unwanted on the test day. However, we want to eliminate any carry-over effect from MDMA to the next test session to avoid elevated duloxetine levels due to MDMA-induced 2D6 inhibition. Therefore, test sessions will be separated by at least 10 days [33] to eliminate potential carry-over effects between sessions. The effect of CYP 2D6 inhibition on the PK of duloxetine has previously been studied and is moderate. Specifically, the maximum plasma concentration of duloxetine and the AUC increased 1.6-fold after pretreatment with the potent CYP 2D6 inhibitor paroxetine [32]. This moderate effect of 2D6 inhibition on the metabolism of duloxetine is explained by the fact that duloxetine is mainly metabolised by CYP 1A2 and only to a minor degree by CYP 2D6 [34].

# STUDY OBJECTIVES

We aim to test the effect of pretreatment with duloxetine on the subjective and cardiovascular effects of MDMA and on the pharmacokinetics of MDMA in humans.

## Primary study endpoints

1. Effect of duloxetine on the subjective response to MDMA

## Secondary study endpoints

1. Effect of duloxetine on cardiovascular effects of MDMA
2. Effect of duloxetine on pharmacokinetics of MDMA
3. Effect of MDMA on duloxetine pharmacokinetics
4. Tolerability of MDMA and duloxetine
5. Effect of duloxetine on neuroendocrine responses to MDMA

# HYPOTHESES

## Primary Hypotheses

1. Duloxetine will attenuate positive mood effects of MDMA. Specifically, co-administration of duloxetine with MDMA will result in a relative decrease in “good drug effects” VAS scores by 20% compared to the MDMA alone condition.

## Secondary Hypotheses

1. Duloxetine will reduce blood pressure and heart-rate increases produced by MDMA.
2. Duloxetine will increase C_max_ and AUC_0-6h_ of MDMA by <30%
3. MDMA will increase C_max_ and AUC_0-6h_ of duloxetine by <30%
4. MDMA and duloxetine will produce no severe adverse effects
5. Duloxetine will decrease neuroendocrine effects of MDMA

# STUDY DESIGN AND METHODS

## Study design

We will use a double-blind cross-over design with four treatment conditions. Treatment conditions are placebo-placebo, duloxetine-placebo, placebo-MDMA, and duloxetine-MDMA. Order will be balanced and pseudo-random. Each subject will participate in four 1-day study sessions with a washout period of two weeks (>10 days) between sessions. We previously used similar designs [18].

## Study duration

1.1.2010 to 31.12.2010

## Study site

Clinical Research Center, Division of Clinical Pharmacology and Toxicology, University Hospital of Basel, Switzerland,

## Study population

### Recruitment

Subjects will be recruited by word of mouth and by advertisements placed on advertising boards of university institutes of the University of Basel and on the homepage of the University. Similar to previous studies [18], we intend to include mainly healthy University students. Six-teen male or female subjects will be enrolled. Drop-outs will be replaced.

### Inclusion criteria

1. Age between 18 and 45 years
2. Sufficient understanding of the German language
3. Subjects understand the procedures and the risks associated with the study
4. Participants must be willing to adhere to the protocol and sign the consent form
5. Participants must be willing to refrain from taking illicit psychoactive substances during the study.
6. Participants must be willing to drink only alcohol-free liquids and no xanthine-containing liquids (such as coffee, black or green tea, red bull, chocolate) after midnight of the evening before the study session. Subjects must agree not to smoke tobacco for 1 h before and 4 hours after MDMA administration.
7. Participants must be willing not to drive a traffic vehicle in the evening of the study day.
8. Women of childbearing potential must have a negative pregnancy test at the beginning of the study and must agree to use an effective form of birth control. Pregnancy tests are repeated before each study session.
9. Body mass index: 18-25 kg/m^2^

### Exclusion criteria

1. Chronic or acute medical condition including clinically relevant abnormality in physical exam, laboratory values, or ECG. In particular: Hypertension (>140/90 mmHg). Personal or first-grade history of seizures. Cardiac or neurological disorder.
2. Current or previous psychotic or affective disorder
3. Psychotic or affective disorder in first-degree relatives
4. Prior illicit drug use (except THC-containing products) more than 5 times or any time within the previous 2 months.
5. Pregnant or nursing women.
6. Participation in another clinical trial (currently or within the last 30 days)
7. Use of medications that are contraindicated or otherwise interfere with the effects of the study medications (monoamine oxidase inhibitors, antidepressants, sedatives etc.)

# STUDY PROCEDURES

## Schedule of Events

The schedule of events for a subject is shown on **table 1**. Subjects take part in a 2 h screening session, four 10 h test sessions, and a 1 h end of study (EOS) visit.

## Screening procedure

### Informed consent

The subjects will be informed about the study both verbally and by the approved written consent form regarding MDMA and duloxetine, the study procedures, and associated risks, in particular including those with the use of MDMA [35]. The investigator and the subject will both personally sign and date the consent form as confirmation of consent.

### Physical health

Subjects will be examined by a research physician. Basic health will be ensured by general medical examination including medical history, physical examination, ECG, and blood chemistry and hematology. Body weight will also be measured.

### Mental health

Subjects will be screened by an MD using a semi-structured clinical interview for DSM IV [36] to exclude those with a personal or family (first-degree relative) axis I major psychiatric disorder or a history of illicit drug dependence.

### History of Drug use

Occasional recreational drug use in the past is not an exclusion criterion if no adverse reactions occurred and if use was moderate and controlled. Drugs screens are primarily intended to document and control for concomitant drug use and not as a means to exclude subjects from the study. However, subjects will be asked to abstain from any illicit drug use during the study and drug screens are performed during screening and before each session. Positive screens for stimulants, opioid or hallucinogens will result in exclusion from the study (next study session). Subjects will also be asked to abstain from excessive alcohol consumption between test-sessions and in particular to limit their use to one glass on the day before the test sessions. Smokers will be asked to maintain their usual smoking habit but will not be allowed to smoke before and up to for four hours after MDMA administration. Positive screens for THC (cannabis) will be recorded but do not result in study exclusion.

### Screening laboratory tests

The following laboratory blood tests are performed at the screening examination: Na, K, creatinine, ASAT, ALAT, γ-GT, hemoglobin, hematocrit, white blood cell count, red blood cell count, platelet cell count. Urine drug screen (Triage-8, Biosite, Morges, CH). Urine pregnancy test.

### CYP2D6 phenotyping

Subjects will be asked to ingest a tablet of dextromethorphan (Bexin®) 25 mg in the evening of the screening day or on a day prior to the test sessions (10-11PM) and to collect and measure the amount of urine during the next 8 h (night urine plus first morning urine). Of this urine two test vials need to be sent to our laboratory in a prepared envelope.

### Genotyping

Polymorphisms in gens that code for monoamine transporters (SLC6A2A and others) contribute to interindividual variations in the clinical response to amphetamines [37]. We will collect a blood sample at the screening visit for genotyping of monoamine transporter genes. In addition genotyping of CYP enzymes are planned (CYP2D6 genotype).

### Personality

Personality traits are known to affect subjective responses to amphetamines [38]. The Multidimensional Personality Questionnaire (MPQ) [39] and the Freiburger Personality Inventory [40] will be used to assess personality traits and their potential modulatory effects on the response to MDMA.

### Menstrual cycle phase in women

Women exhibit differential subjective effects to amphetamines during the follicular as compared to the luteal phase of the menstrual cycle [41]. Therefore, female subjects will preferentially be tested during the follicular phase (day 2-14) of the cycle.

## Schedule of experimental session

**Table 2** shows the schedule of events for each experimental session. Each of the four test sessions will last 10 h from 8:00 AM to 6:00 PM. Subjects will arrive at 8:00 AM at our CRC. Subjects will take duloxetine (120 mg) or placebo the night before the study session at 8:00 PM and another dose of duloxetine (120 mg) or placebo in the morning of the study day (8:00 AM). An indwelling intravenous catheter will be inserted into a subcutaneous vein of the forearm of the nondominant arm. MDMA (125 mg) or placebo is administered 4 h after duloxetine (12:00 AM). The acute effects of MDMA are expected to last for 4 h [42, 43]. Outcome measures (see below) will repeatedly be assessed during the study session. The sessions end at 6 PM. Subjects will be under continuous medical control until the effects of the drug have completely subsided (within 4-6 h after MDMA administration).

## Assessments and Measures

### Psychometric assessments

#### Visual Analogue Scales (VAS)

Previously tested and sensitive VAS will be repeatedly used to better characterize subjective drugs effects over time [16, 44-46]. VAS will be presented as are 100 mm long horizontal lines marked with “not at all” on the left and “extremely” on the right [46]. The following VAS will be used: “drug effect”, “good effect”, “bad effect”, “liking”, “high”, “energy level”, “feelings of closeness to others”, “mind racing”, “ability to concentrate”, “fear”, “hungry”, “sedated”, “talkative”, “open”, and “confused”. Scales will be administered 4 h before and 0, 20, 60, 90, 120, 150, 180 min, and 4, 5, and 6 h after MDMA/placebo administration [45].

#### Adjective mood rating scale (AMRS)

An adjective mood rating scale (AMRS or EWL60S) is a 60-item Likert scale that allows repeated assessment of mood in 6 dimensions: activation, inactivation, well-being, anxiety/depressed mood, extro- and introversion, and emotional excitability. The scale is sensitive to the effects of MDMA [13, 24, 42, 47]. It will be used before, 75, 120 min and 5 h after MDMA administration. The German EWL60S version is used [48].

#### State-trait anxiety inventory (STAI)

The state-trait anxiety inventory (STAI) yields a score for state and trait anxiety levels [49]. The trait scale will be administered once. The state anxiety scale will be used before, 75, 120 min and 5 h after MDMA administration.

#### Addiction Research Center Inventory (ARCI)

The Addiction Research Center Inventory (ARCI) is a true-false questionnaire with five empirically derived scales: pentobarbital-chlorpromazine-alcohol group, a measure of sedation; morphine-benzedrine group, a measure of euphoria; lysergic acid diethylamine group (LSD), a measure of dysphoria and somatic symptoms; benzedrine group, a stimulant scale consisting mainly of items relating to intellectual efficiency and energy; and amphetamine, an empirically derived scale sensitive to the effects of d-amphetamine [50]. It has previously been shown to be sensitive to the effects of MDMA [16, 17] and is available in a validated German version [51]. The ARCI will be administered 4 h before and 150 min after MDMA administration.

#### Altered states of consciousness (5D-ASC)

The 5 dimensions of altered states of consciousness (5D-ASC) scale is a visual analogue scale consisting of 94 items [52]. The instrument is constructed of five scales and allows assessing mood, anxiety, derealization, depersonalization, changes in perception, auditory alterations, and reduced vigilance. The scale has repeatedly been used to evaluate subjective responses to MDMA [24, 42, 47] or other psychoactive substances [53]. The 5D-ASC scale will be administered once 4 h after MDMA administration to retrospectively assess drug peak effects.

### Physiological assessments

#### Vital signs

Blood pressure, heart rate, body temperature, and pupil diameter will be recorded at -4 h, 0, 20, 40, 60, 90, 120, 150, 180 min, and 4, 5, and 6 h after MDMA/placebo administration. Blood pressure (systolic and diastolic) and heart rate will be measured on the leading arm using an automatic oscillometric device. Heart rate will also be measured continuously with a RS 800 Polar device (Polar Electro Oy, Kempele, FIN), set to sample the average heart rate every 5 s, and analyzed using Polar Precision Performance software. Body temperature will be measured using an ear thermometer. Ambient temperature is also recorded trice on the experimental day. Pupil diameter and the reaction to a light stimulus will be recorded using a handheld dynamic pupillometer (PRL-200, Neuroptics, Irvine, CA, USA).

#### List of complaints (LC) and Adverse effects (AE)

Adverse effects (AE) will be assessed systematically during the session using a modified list of complaints (LC) [54]. The scale consists of 66 items, yielding a global score measuring physical and general discomfort. The LC list is administered 4 h before and 180 min after MDMA/placebo. An additional LC is completed 24 h after MDMA/placebo in the absence of the experimenter. Additional AE during the session will be listed in the case report form (CRF) 5 h after MDMA/placebo administration. Subjects will also be asked to report AE between study sessions at the beginning of the next session.

### Cognitive and psychomotor function

#### Reading the mind in the eyes test (RME)

A 10-min computerized “Reading the Mind in the Eyes Test” is performed 90 min after MDMA/placebo administration (before the CRTT). The test assesses drug effects on inferring mental states from the eye region. 36 pictures of the eye regions of different persons will be presented to the participants on a computer screen with four alternative labels describing what the person displayed might be thinking or feeling at the moment [55].

#### Choice reaction time test (CRTT)

This five choice reaction time test (CRTT) assesses a combination of sustained attention and executive motor function [56]. The CRTT will last for 3 min and requires the subject to quickly and accurately press a button of the same color as the one of five (red, blue, white, yellow, or green) which flashed. The stimuli are presented in random sequence. Using a computer-based algorithm, the interstimulus interval is adjusted by 50 ms according to performance in a continuously moving window of the last seven stimulus–response pairs. The interstimulus interval decreases when four or more stimulus–response pairs were correct; otherwise, the interval increases. Thus, test difficulty is adjusted to achieve a subject false response rate of 50%, which makes for a demanding task. The dependent variable is the mean reaction time (for correct responses) for the last 2 min of the test. Reaction times less than 300 ms will be considered artifactual and are therefore discarded. Subjects will go through one training session at the beginning of each session. Tests will then be performed 0 min and 90 min after MDMA administration.

#### End of Session Questionnarie

At the end of each session, participants will be asked to indicate how much they liked the drug overall and how strong the overall effect was on separate VAS [16].

### Neuroendocrine function

MDMA increases cortisol, prolactin, catecholamine, and 5-HT concentrations in plasma in humans [43, 57-59]. MDMA also increases vasopressin (AVP) potentially leading to hyponatriemia [59, 60]. We will test the effect of duloxetine on MDMA-induced increases in plasma cortisol, prolactin, catecholamines, serotonin, and metanephrines (norepinephrine and epniephrine metabolites). Plasma cortisol and prolactin samples will be collected at 0 min and at 120 min (t_max_) after MDMA/placebo administration (2x7.5 ml blood). Copeptin is the C-terminal part of provasopressin. Copeptin is released stoichiometrically together with AVP and is easier to measure than AVP. Copeptin samples will be collected at 0 min and 120 min after MDMA/placebo administration when peak AVP values are expected [59] (2x7.5 ml blood). Plasma and urine sodium, urea, and osmolality are simultaneously assessed (2x7.5 ml blood, 2x10 ml urine). Catecholamine and metanephrine samples will be collected at 0, 60, and 120 min after MDMA/placebo administration (3x7.5 ml blood).

### Blood sample collection (pharmacokinetics)

Blood samples (11x7.5 ml) will be collected on the study day -4, 0, 0.33, 0.67, 1, 1.5, 2, 2.5, 3, 4, and 6 h after MDMA or placebo administration. This will allow us to assess the pharmacokinetics of both MDMA and duloxetine and potential PK interactions of the two compounds. The design is optimized to assess effects of duloxetine on peak plasma levels of MDMA (time to C_max_ = 2h) and AUC_0-6h_ for MDMA [43]. Blood samples will be collected in heparinized tubes, centrifuged at 4°C at 2000 rpm for 10 min, and the plasma is then stored at -20°C (two aliquots) until analysis. Collection tubes labels will include the following information: study number, study day, subject number, and sample number. The date and actual blood sampling time will be recorded on the sample collection page in the CRF.

#### Amount of blood samples

On each study day a total of 20 blood samples (20x7.5 ml = 150 ml) will be collected on the study day. During the whole study approximately 600 ml of blood are collected. This amount to 20% more than a standard blood donation. However, blood is collected in amounts of 150 ml every 2 weeks spread out over 6 weeks and not at once which allows for regeneration. In order to blind the study, plasma samples will be collected also on the placebo day.

### End of Study (EOS) visit

#### EOS Examination

Clinical examination and vital signs. Blood tests: Na, K, creatinine, ASAT, ALAT, γ-GT, hemoglobin, hematocrit, white blood cell count, red blood cell count, platelet cell count. Adverse effects.

#### EOS Questionnaire

A debriefing interview will be performed including a retrospective comparative evaluation of the subjective experience of all four study sessions. Subjects will be asked to rate the overall drug effect for each of the four sessions. Subjects will also be asked to guess the assignment of the study drugs to the four study sessions to assess whether subjects were able to distinguish the study conditions (duloxetine vs. placebo). They will also be asked to provide a feed-back on the study and their experience and to indicate whether they would take the drug again in a controlled or recreational setting [15].

## Study Drugs

### Duloxetine

Duloxetine (Cymbalta) is a potent and selective 5-HT and NE reuptake inhibitor [27]. Duloxetine (60 mg gelatine capsules) will be obtained from Eli Lilly (Suisse) SA, Vernier. The investigator pays for the product. Eli Lilly does not act as a sponsor for the study. The original company product will be used. However, capsules will be encapsuled with opaque dark-colored gelatine capsules and similar placebo capsules containing lactose will be prepared by a pharmacist. **Clinically used doses:** Duloxetine is used clinically in the treatment of depression, generalized anxiety disorder, diabetic and other neuropathy, and fibromyalgia. The clinically used common starting dose is 60 mg/day. Maintenance doses are 60-120 mg/day [25, 61, 62]. Several clinical trials have used 120 mg/day given once or in two doses of 60 mg twice per day. The suggested maximum dose is 120 mg daily. **Tolerability:** Duloxetine was well tolerated at doses of 60-120 mg/day [63]. See 14.1. for expected adverse effects of duloxetine. **Pharmakokinetics (dose finding and dosing intervals):** Elimination half-life of duloxetine is 12 h and T_max_ is 6 h [61, 64]. On the test day, duloxetine (120 mg) will be given at 8 AM 4 h before MDMA administration at 12 AM so that the duloxetine C_max_ will be reached shortly after the onset of the subjective MDMA effect (1h after MDMA administration) and together with C_max_ of MDMA. An additional dose of duloxetine (120 mg) will be administered the day before MDMA at 8 PM, 16 h before MDMA administration. At both time-points duloxetine will be administered in a dose of 120 mg (maximum suggested therapeutic dose). Because the study uses only one dose of duloxetine it is important to use a relatively high dose to ensure effective plasma levels. Mean duloxetine plasma C_max_ values were 22, 44, and 61 ng/mL after single oral doses of 30, 60, and 90 mg of duloxetine in healthy volunteers [64]. At steady state 40 mg once daily resulted in a mean C_max_ level of 35 ng/mL and a trough level of approximately 10 ng/mL at 24h [32]. Population PK studies showed mean C_max_ levels of 30-60 ng/mL follwing daily administration of 60 mg and depending on gender, smoking status, age and other variables [65]. Mean trough levels at 24h were in the range of 25 ng/mL. Women typically had 64% higher average steady-state concentrations than men (with considerable overlap). Similarly, nonsmokers had 43% higher average concentrations than smokers. Doubling the duloxetine dose from 30 mg to 60 mg and from 60 mg to 120 mg resulted in 2.3 fold and 2.6 fold higher average concentration values, respectively [65]. Based on these data we expect mean C_max_ levels of 120-150 6 h after the administration of the second dose of 120 mg of duloxetine with the first dose (120 mg) given 12 h before the second dose. This C_max_ is similar to the one seen in patients treated with daily doses of 120 mg under steady state conditions (peak 100 ng/mL, trough 50 ng/mL) [65]. **PD and PK interactions of duloxetine and MDMA** (see 7.2.1/2). **Relevant clinical studies using duloxetine in healthy volunteers**: Duloxetine (60 mg for 14 days) was not able to block the thyramine-induced increases in systolic blood pressure, indicating that the dose might be too low to block the NE transporter to a relevant extent in this test [66]. A dose of 80-120 mg was effective in suppressing the blood pressure increase due to thyramine [25]. However, there is clinical evidence that lower doses of duloxetine block NE transport. For example, urine catecholamine changes indicative of NE reuptake inhibition were present already at 80 mg. We suggest that a dose of 120 mg should be used for the present study. Of note, desipramine completely blocked thyramine-induced SBP responses which could indicate that desipramine interacts with stimulants at a different site on the NE transporter than duloxetine.

### MDMA

#### Chemistry, manufacturing, control

Pharmaceutically pure racemic MDMA HCl (±3,4-methylenedioxymethamphetamine hydrochloride) (Lipomed AG, Arlesheim, Switzerland) will be used similar to previous studies [47, 67, 68]. Quality control of the drug confirmed purity (>98%) and content of the batch and absence of decomposition products. MDMA from this lot has previously been used in human studies [67]. MDMA from the same batch is also being used in clinical trials in several countries including Switzerland [69]. MDMA will be prepared as capsules (100 mg and 25 mg) by a BAG authorized laboratory according to good manufacturing practice (GMP) criteria. Identically looking placebo capsules will contain lactose and will be provided by the same laboratory. An updated quality control certificate for the final study drug preparations and all labeling information will be provided before study initiation.

#### Metabolism and Pharmacokinetics

After a single dose of MDMA t_max_ is attained at 120-144 min [44, 70]. T_1/2_ of MDMA (100 -125 mg dose) is about 8-9 h [71]. MDMA inhibits its own metabolism possibly by forming an enzyme-metabolite complex with CYP2D6. This autoinhibition likely explains the suggested non-linear pharmacokinetics of MDMA [33, 70-72]. There are two main metabolic pathways for the metabolism of MDMA [71]. MDMA is N-demethylated to 3,4-methlyenedioxyamphetamine (MDA). MDA is an active, but minor metabolite. CYP2D6-mediated O-demethylenation followed by COMT-catalyzed methylation leads to 4-hydroxy-3-mehoxymethamphetamine (HMMA) or 4-hydroxy-3-methoxyamphetamine (HMA). HMMA is the main metabolite of MDMA [71]. HMMA and HMA are rapidly glucuronidated or sulphate-conjugated.

#### Dose selection

An oral dose of 125 mg MDMA corresponding to about 1.5-1.7 mg/kg will be used similar to previous studies [42 , 44, 46, 47, 58, 70]. This dose of pure MDMA produces moderat subjective and cardiovascular effects [24, 42, 47, 67] and is considered safe in a controlled clinical setting [2, 35, 70, 73]. The same dose of 125 mg followed 2.5 h later by 62.5 mg MDMA is currently being used in a clinical study [69]. Higher doses (150 mg or 2 mg/kg) have also been used without adverse events in MDMA studies [42, 69, 74, 75] and are used in clinical studies as well (150 mg plus 75 mg 2.5h later). Ecstasy tablets usually contain 80-160 mg of MDMA [76]. Use of more than one tablet at once is common.

### Drug-Accountability, storage and return of study drugs

The study drugs will be stored in a locked drug cabinet in a lockable room with restricted access at room temperature (15-25°C) at the clinical research center. A drug dispensing and accountability log for all capsules will be kept current. The log includes the date, origin, and amount of incoming study drug. The study physician/investigator who dispenses the drug, registers and signs out the drug quantity, dispensing date of dispensing, participant number for all outgoing medications.

### Concomitant medications

Concomitant use of medications will be recorded at the screening visit and at the beginning of each study session. Subjects are asked to abstain from using drugs that may interfere with the effects of the study medications including sleeping aids, cough medications, betablocker or other substances with potentially relevant psychoactive effects. The PI may exclude subjects from study participation or reschedule sessions in the case of relevant pharmacokinetic of pharmacodynamic interactions with concomitant medications.

### Randomization and Blinding

Each subject will receive all treatments. Subjects and study personnel will be blinded to treatment order. Order is balanced and pseudo-random. A study subject number will be assigned to each subject. A treatment order is assigned to each subject number (code). Treatments are prepared and labeled according to the code by a pharmacist not involved in the study. The PI will also receive the code enclosed in an envelope. In case of a medical emergency the code can be accessed.

### Compliance

Subjects are provided with the duloxetine medication box for each session and are reminded to take the medication by a phone call the day before the study session. Compliance is assessed by asking the subject to report the time of drug intake, by collection of the empty medication box, and by determination of duloxetine in plasma (PK sample -0 and 1). During the study day medications are administered under observation by the study personnel.

# Analytics

## MDMA and metabolites

MDMA, its active metabolite MDA and the main inactive metabolites HMMA and HMA will be determined in plasma by gas chromatography-mass spectrometry (GC-MS) by established techniques as previously described [77, 44, 78].

## CYP2D6 Phenotype

Dextrorphan/dextromethorphan ratio will be used for CYP2D6 phenotyping [79, 80].

## Cortisol and prolactin

Plasma cortisol and prolactin concentrations will be determined by standard immunoassay techniques.

## Catecholamines and metanephrines

Plasma metanephrines, catecholamines, and 5-HT will be measured by HPLC with electrochemical detection at the University Hospital Lausanne.

## Copeptin

Plasma copeptin levels will be measured with a sandwich immunoassay (BRAHMS AG, Hennigsdorf, Berlin, Germany) [81].

## Duloxetine

Analysis of duloxetine plasma levels will be performed using LC-MS-MS.

# Data analysis

## Sample size estimation

Power analysis was performed with PASS®, Hintze J. Kaysville, Utah, US. We consider a relative reduction of 20% (MDMA vs. duloxetine+MDMA) in “good drug effects” to be clinically meaningful [13, 16, 17, 68]. A sample size of 13 achieves 82% power to detect a difference of 20 between the null hypothesis mean of 100 and the alternative hypothesis mean of 80 with a known standard deviation of 25 [13, 68] and with a significance level (alpha) of 0.05 using a two-sided one-sample t-test. For the most relevant secondary outcome, which is P_sys_, we expect that duloxetine will decrease the MDMA-induced increase in P_sys_ by 10 mmHg from +30 mmHg (MDMA vs. placebo) to +20 mmHg MDMA+duloxetine vs. duloxetine). This effect of duloxetine would be relevant and similar to the one of citalopram on MDMA-induced increases in P_sys_ [15]. A sample size of 8 achieves 81% power to detect a difference of 10 between the null hypothesis mean of +30 and the alternative hypothesis mean of +20 with a known standard deviation of the difference of 10 [15] and with a significance level (alpha) of 0.05 using a two-sided one-sample t-test. Assuming a drop-out rate before inclusion of 50% we will have to screen 32 subjects to include 16 in the study. Lower (8-12) [16, 17] or similar sample sizes [13, 47, 68, 82] were used in previous comparable studies. Subjects who prematurely discontinued the study for any reason will be replaced if the number of discontinued subjects exceeds three. A samples size of 8 or less is commonly used in PK studies. A larger sample is used here to produce meaningful results for the pharmacodynamic parameters.

## Analysis of outcomes

Repeated-measures analysis of variance (ANOVA) with drug (MDMA and duloxetine) as within-subject factors followed by Tukey’s post-hoc tests will be use to analyze differences between the different treatment conditions. Transformations will be performed before analysis, if response variables are nonnormally distributed. Pharmacokinetic parameters (C_max_, t_max_, AUC from 0 to 6 h) will be obtained using *PK Functions for Excel*®. AUC values will be calculated by the trapezoidal rule. Additional PK analyses may be performed using *WinNonLin®* and *GraphPad®.* Statistical analyses will be performed using *Statistica*® *(StatSoft).*

# Protection of subjects

Trained study personnel will place venous accesses, administer the study drugs and collect blood samples. Subjects will be supervised by trained study personnel during the entire test sessions, backed-up by study physicians. After the test session subjects are allowed to leave only if the subjective and cardiovascular effects of MDMA have completely ceased (see 14.2.5). In the case of unexpected prolonged or severe adverse reactions subjects will remain at the CRC and are monitored as long as necessary. Subjects will be provided with the phone number of the study physician on call in case of an emergency between/after study sessions.

## Potential risks

Physical risks:

MDMA: A large series of phase I like studies have been performed using MDMA [13-16, 24, 42, 44-47, 57, 58, 67, 68, 70, 71, 74, 75, 82-91]. This considerable body of evidence indicates that the likelihood for significant toxicity from doses of MDMA such as the one to be used in the present study and in controlled settings is very low. Thus, no lasting biological or psychological injury is expected. See below for specific MDMA toxicity and monitoring. Expected adverse effects of MDMA: MDMA is expected to produce moderate acute adverse effects such as increased jaw muscle tension, dry mouth, dizziness, palpitations, tremor, nausea, headache, insomnia, and transient anxiety in more than 10% of subjects, [15, 42]. MDMA is expected to produce sequelae (1-3 days post MDMA) including difficulty concentrating, irritability, or slightly depressed mood in more than 10% of subjects [15]. No severe or serious adverse effects are expected. Duloxetine: In placebo-controlled double-blind trials in patients duloxetine (60-120 mg/day) produced nausea (20-29%), headache (20%), dry mouth (15-18%), insomnia (11-15%), fatigue (14%), dizziness (9-11%), constipation (11%), decreased appetite (8%), and sweating (6%) [63]. Adverse effects of duloxetine in healthy volunteers similary include nausea, dry mouth, insomnia, dizziness, sweating, and clinically non-significant increases in blood pressure or heart rate [25, 61, 92]. No severe or serious adverse effects are expected.

Venipunction: There is a risk for pain, bruising and thrombophlebitis.

Risk to privacy of subjects: Potential risks of data collection include breach of confidentiality. Financial risks: There is no risk of expense to the subject. Insurance coverage is provided. Travel expenses are expected to be minimal and are included in the compensation of 1000 CHF per subject.

## Monitoring of toxicity

### Safety definitions

Adverse Event (AE):

Any untoward medical occurrence in a clinical trial subject administered an investigational medicinal product (IMP) and which does not necessarily have a causal relationship with this treatment. An AE can therefore be any unfavorable and unintended sign (including an abnormal laboratory finding), symptom, or disease temporally associated with the use of an (IMP), whether or not considered related to the IMP.

Adverse Reaction (AR):

All untoward and unintended responses to an IMP judged by investigator/sponsor as having a reasonable causal relationship to the IMP. The expression reasonable causal relationship means to convey in general that there is evidence or argument to suggest a causal relationship.

Unexpected Adverse Reaction (UAR):

An AR, the nature or severity of which is not consistent with the applicable product information (e.g. investigator’s brochure for an unapproved investigational product or summary of product characteristics (SmPC) for an authorized product). When the outcome of the adverse reaction is not consistent with the applicable product information this adverse reaction should be considered as unexpected. Side effects documented in the IB or SmPC which occur in a more severe form than anticipated are also considered as being unexpected.

Serious Adverse Event (SAE) or Serious Adverse Reaction:

Any untoward medical occurrence or effect that at any dose results in death, is life-threatening, requires hospitalization, or prolongation of existing hospitalization, results in persistent or significant disability or incapacity or is a congenital anomaly or birth defect. In this context, the term life threatening refers to an event in which the trial participant was at immediate risk of death at the time of the event; it does **not** refer to an event, which might have caused death if it were more severe.

Suspected Unexpected Serious Adverse Reaction (SUSAR):

Any suspected adverse reaction related to an IMP that is both unexpected and serious.

**Causality:**

Most adverse events and adverse reactions that occur in this study, whether they are serious or not, will be expected treatment-related toxicities due to the medication used in this study. The assignment of the causality will be made by the investigator using the definitions in the table below.

| **Relationship** | **Description** |
| --- | --- |
| **Unrelated** | There is no evidence of any causal relationship. |
| **Unlikely** | There is little evidence to suggest there is a causal relationship (e.g. the event did not occur within a reasonable time after administration of the IMP). There is another reasonable explanation for the event (e.g. the participant’s clinical condition, other concomitant treatment). |
| **Possible** | There is some evidence to suggest a causal relationship (e.g. because the event occurs within a reasonable time after administration of the IMP). However, the influence of other factors may have contributed to the event (e.g. the participant’s clinical condition, other concomitant treatments). |
| **Probable** | There is evidence to suggest a causal relationship and the influence of other factors is unlikely. |
| **Definitely** | There is clear evidence to suggest a causal relationship and other possible contributing factors can be ruled out. |
| **Not assessable** | There is insufficient or incomplete evidence to make a clinical judgment of the causal relationship. |

### Documentation

All adverse events occuring after the subject has signed the informed consent will be fully recorded in the subject’s CRF. Each event will be described in detail along with start and stop dates, severity, relationship to investigational product, action taken and outcome.

### Adverse events (AE) documentation

AE events will be described and recorded on the subject’s CRF, regardless of the severity or relationship to the drug. Adverse effects are assessed by the standardized list of complaints (LC) [54]. Additional AEs are entered in the CRF at the end of the study session. AEs are also assessed 24 h after MDMA/placebo using the LC. Further, AEs are assessed before the next session 2 weeks after MDMA/placebo. AEs are rated for severity and the potential relationship to the study medications will be evaluated by the investigator according standard criteria. Subjects with AEs will be treated appropriately. Abnormal laboratory values will be repeated until normal or until the abnormality can be explained and the subject’s safety is not at risk. In cases of a medical emergency, treatment is available in house and includes the 24h-availability of the reanimation team and intensive care facilities.

### SAE and SUSAR reporting

Serious adverse events (SAE) are not expected. Should a SAE occur the PI will be informed and relationship to the study drug will be assessed by the PI using the definitions shown above (14.2.1.). Complete information concerning the SAE will be collected by the PI and documented on a standard SAE form. Any SUSAR will be reported to the IRB and Swissmedic by the PI according to guidelines and within 7 days. The PI acts as sponsor in this investigator-initiated study. Therefore no reporting to a sponsor is needed. An annual safety report will be sent to Swissmedic by the PI.

### Medical follow-up of adverse events

The investigator will ensure the subject receives medical follow-up as necessary until the condition has stabilized or returned to normal state, even if the period of the trial is over.

### Monitoring for specific toxicity

Duration of monitoring: Subjective and cardiovascular effects of MDMA last for a mean of 3.5 h and completely subside within 5-6 h [13, 42, 43, 58, 67]. Accordingly subjects are monitored for 6 h after MDMA administration. In our previous studies, that similarly evaluated interactive effects of MDMA with various pretreatments, subjects were dismissed 4 h after MDMA administration [13, 15, 68, 82]. Another interaction study used an observation time of 6 h after MDMA administration similar to the present study [43]. Only one to two subjects are tested simultaneously on the same test day.

Cardiovascular effects: Blood pressure and heart rate are measured at repeated intervals (Table 2). Closer monitoring will be implemented if blood pressure values exceed 180/120 mm Hg. Subjects will be monitored for signs of hypertensive crisis such as headache, angina pectoris or neurological deficits. Treatment of a hypertensive reaction (P_sys_>220 mmHg) would include administration of oxygen, lorazepam, nitroglycerin or nifedipin or/and carvedilol. In the case of angina pectoris, oxygen is given and ECG monitoring is initiated and cardiac enzymes will be measured. Subjects will remain at the CRC overnight for further observation if blood pressure is >140/95 mmHg or pulse rate is >100/min at the end of the test session at 6 PM.

Psychological effects: The effects of MDMA are mostly pleasurable. Dysphoric reactions to MDMA and moderate anxiety may occur at the onset of the MDMA effect. Such effects have always resolved within hours [42]. Unexpected severe anxiety would be treated with a benzodiazepine. Up to one third of the subjects is expected to experience slightly depressed mood including emotional irritability, lack of energy, brooding and bad dreams for up to 3 days following MDMA administration [42]. These effects will be specifically monitored using an adverse effects check list. After the test session subjects are allowed to leave only if the subjective effects of MDMA have completely ceased (VAS score “drug effect” back to predose ± 10%).

Hyperthermia: Increases in body temperature of approximately 0.5°C are expected [42]. If body temperature rises by more than 1.5°C appropriate treatment including hydration and cooling will be initiated [5]. Subjects will remain at the CRC overnight for further observation if body temperature has not returned to pretreatment levels ± 0.5 °C at the end of the test session at 6 PM.

Dehydration/Hyperhydration: Ecstasy use has been associated with both dehydration and hyperhydration leading to hyponatremia [5]. Subjects will be encouraged to drink no less than 1 but no more than 3 l during one session. If symptoms of hyponatremia such as confusion or vomiting are present, subjects will be further examined and serum sodium levels checked.

Hepatotoxicity: Ecstasy use has been associated with idiosyncratic liver toxicity [5]. Liver enzymes will be measured at the beginning and end of the study and any pathological results will be followed up.

Neurotoxicity: Recreational use of Ecstasy has been linked to reversible neurotoxicity [93, 94], especially when the drug is taken frequently and in high doses [6, 93]. In contrast, several studies document no lasting effects of Ecstasy in moderate Ecstasy users [93, 95-97]. There is no indication that administration of MDMA in a controlled setting has any adverse effects on cognitive function [2, 89, 98]. The use of pure MDMA in controlled settings is considered safe with regard to neurotoxic effects [35, 73]. No changes in serotonin transporter density were seen using positron emission tomography and [11C]-McN5652 four weeks after MDMA administration (1.5-1.7 mg/kg) in MDMA-naïve human volunteers [99, 100]. We will administer only two single moderate doses of MDMA similar to previous clinical studies [13, 16, 17, 47, 67, 68, 82].

Abuse liability: The likelihood for future drug abuse triggered by the administration of MDMA in a controlled setting is assumed to be very low. MDMA has rewarding effects in animals and humans [74] but the risk for developing dependence is considered low when compared with other drugs of abuse [101]. In previous studies using MDMA in controlled settings, none of the participants expressed any interest in taking MDMA as a recreational drug after completing the study [15, 42]. Subjects with known risk factors for future MDMA use including depression or anxiety disorders [102] and prior drug dependence or other relevant illicit drug use [103] will be excluded from the study. Illicit drug use will also be monitored during the study using repeated urine drug screens.

## Risk-Benefit Assessment

Potential benefits: First, the present study will generate objective, scientific information about the effects of MDMA that could not be obtained with observational studies. MDMA is used non-medically by millions of people and scientific information on the pharmacological and toxicological effects of MDMA is needed. Second, research into the mechanisms of action of MDMA, which has strong mood-enhancing effects, will provide insight into the physiology of mood and pathophysiological processes involved in mood disorders. Third, normative data on the effects of MDMA in normals are useful to interpret results form studies in Ecstasy users. Forth, pharmacodynamics and pharmacokinetics of MDMA should be better characterized in the light of ongoing clinical phase II trials using similar doses of MDMA. The importance of these issues is recognized by researchers and regulatory authorities as demonstrated by the increase in number of approved controlled studies in humans.

Risk/benefit Ratio: Overall, the risk for severe adverse effects or lasting physical harm is considered very low. With regard to subjective distress, the overall effect of MDMA is clearly a pleasurable experience with comparatively minimal risks for dysphoric reactions and tolerable adverse effects. The potential risk of further abuse of MDMA is expected to be very low in healthy subjects with low risk of drug abuse liability such as previous significant drug use or preexisting psychiatric disorder. Taken together, the risks are minimal. Because healthy volunteers will participate in this phase I study, the benefits mainly consist in increased information that will not directly benefit the study participants but will be of benefit for the society and patients with Ecstasy intoxications. However, based on our experience with similar studies, subject mostly experienced pleasure and considered the use of MDMA in a controlled setting to be of some interesting and personal value.

## Ethical standards

The study will be performed in accordance with the Declaration of Helsinki, International Conference on Harmonization, Topic E6 Guidelines for Good Clinical Practice (GCP), the “Verordnung über klinische Versuche mit Heilmitteln” (VKlin), the “Heilmittelgesetz” (HMG), and standard operating procedures of the CRC/CTU of the University Hospital of Basel.

### Institutional Review Board

The PI will submit the study protocol and informed consent form to an approved IRB (institutional review board = Ethische Kommission beider Basel, EKBB).

### Protocol amendments

Any protocol amendments will require formal approval of the ethics committee prior to their implementation.

### Early study termination

The study may be ended early in the case of unexpected adverse events or other reasons based on the PIs decision and on criteria determined as outlined below.

- An interim analysis will be performed after 8 subjects have completed the study. If this analysis indicates a significant increase in measures that indicate increased monoamine release the study will be terminated early. Measures included in the analysis are: heart rate, blood pressure, the total of adverse effects (list of complaints), and adverse events.
- In addition, in the case of severe adverse events in a subject indicating excessive monoamine release, the code will be opened and results analyzed by the principal investigator. The following findings when due to MDMA and duloxetine administration will trigger early analysis of results and potential study termination:
  - Hypertension >P_sys_ 200 mm Hg or P_dia_ 140 mm Hg
  - Tachykardia 120 beats/min
  - Serotonin syndrome (cloni, hyperthermia, delir)
  - Other unexpected severe adverse effects that could be linked to excessive release of monoamines

The PI will inform the IRB and Swissmedic when the study ends within 90 days or 15 days if the study is terminated early. A final report is sent to both within six months of study termination by the PI.

### Insurance

Subjects will be insured for this trial by Zurich Insurances via the University Hospital. The PI will be responsible for the initiation of all necessary action in the case of harm to a subject.

### Compensation

Subjects will receive a financial compensation of 1’000 CHF for the completed trial. Pro rata payments will be done for early discontinuation. There are no other compensations.

### Premature withdrawal of subjects

Subjects have the right to withdraw from the study at any time. The investigator may withdraw subjects from the study in the event of adverse events, protocol violations, or other reasons. An end of study assessment is made at the time of the subject's withdrawal. If the reason for removal of a subject from the study is an adverse event or an abnormal laboratory test result, the event or test will also be recorded on the CRF.

### Replacement policy

Subjects who withdraw from the study will be replaced if the number exceeds three subjects, to maintain at least 13 subjects. Replacement subject will follow the same treatment sequence as the withdrawn subject.

## Legal authorizations

MDMA is a scheduled illegal substance in Switzerland (Anhang d der BetmV-Swissmedic). We will apply to the BAG for permission to use MDMA in the present study as soon as the study has been assessed by the IRB and Swissmedic.

## Trial registration

The study will be registered at [www.clinicaltrials.gov](http://www.clinicaltrials.gov/).

## Study documentation and record keeping

The investigator will maintain adequate records to enable the conduct of the study to be fully documented. Copies of protocols, CRFs, originals of test result reports, drug dispensing logs, correspondence, records of informed consent and other documents pertaining to the conduct of the study will be kept on file for 10 years in the CRC archive. Identification codes will be kept for at least two years after study completion. All forms should be typed or filled out using a blue or black ball-point pen, and must be legible. Errors should be crossed out but not obliterated, the correction inserted, and the change initialed and dated by the investigator or an authorized person. For each subject enrolled a CRF will be completed and signed by the investigator. This also applies to those subjects who fail to complete the study.

## Quality control and quality assurance

### Training of personnel and SOPs

The study is performed in accordance with ICH GCP E6 and the SOPs of the phase 1 unit and the study coordination center of the clinical trial unit (CTU) Basel. The study personnel has completed a clinical investigator course of the CTU Basel and additional on-site training.

### Monitoring

The trial will be monitored by the CTU Basel to verify the accuracy and completeness of CRFs, to ensure that all protocol requirements, applicable local authority regulations and investigator’s obligations are being fulfilled, and to resolve any inconsistencies in the study records.

### Direct access to source data

The investigator will permit study related monitoring visits, audits, ethics committee reviews, and regulatory inspections, and provide direct access to all source data. Source data are all information, original records of clinical findings, observations, or other activities in a clinical trial necessary for the reconstruction and evaluation of the trial.

### Inspection

Inspections by regulatory authorities during study or after study closure might be performed to ensure proper study conduct and data handling procedures according to ICH-GCP guidelines and regulatory requirements. Inspections may include verification of all source documents, check of CRFs and site files and a visual inspection of the study site.

## Confidentiality of trial documents and subject records

The investigator will assure that subjects' anonymity will be maintained. On CRFs subjects should not be identified by their names, but by an identification code. The investigator will keep a separate log of subjects' codes, names and addresses.

# Time plan

Start of the study: 1.1.2010

Study duration per subject: 7 weeks

Study completion: 31.12.2010

Final report: 31.3.2011

# Publications

All results will be presented at a congress meeting and published in a peer-reviewed scientific journal. The PI will be senior and corresponding author on all publications. PhD candidates who will substantially contribute to the work will be first authors.

# Budget and Funding

The costs will be covered by a grant of the Swiss National Science Foundation (SNF). The researchers do not receive any payments for the conduct of this research besides from their salaries.

# Certifications of principal investigator

The PI is a MD specialized in internal medicine and clinical pharmacology and toxicology. He holds a MAS degree in Clinical Research (2005-2007, UCSD, CA, US). He has successfully been conducting clinical research and similar studies using MDMA in healthy volunteers [13-15, 18, 24, 42, 68, 82, 104] and works as senior physician in the Clinical Pharmacology and Toxicology Unit.

# Conflict of interest

None

# Research Environment

## Study site

The study will take place in the fully equipped eight-bed Clinical Research Center of the University Hospital Basel, Switzerland. This controlled setting allows for close monitoring of adverse effects and know-how and staff for PK sampling is available.

# References
